# Delaying Stress Granule Disassembly by PARG Inhibition Attenuates Renal Tubular Cell Pyroptosis in Acute Kidney Injury

**DOI:** 10.3390/ijms27167192

**Published:** 2026-08-12

**Authors:** Yiyun Song, Shan Jiang, Min Yang, Jinchai Zhu, Banghuan Hu, Hua Su

**Affiliations:** 1 Department of Nephrology, Union Hospital, Tongji Medical College, Huazhong University of Science and Technology, Wuhan 430022, China; 2 Department of Nephrology, The First Affiliated Hospital of Dalian Medical University, Dalian 116011, China; 3 Department of Nephrology, Chongqing Hospital Union Hospital, Tongji Medical College, Huazhong University of Science and Technology, Chongqing 401121, China

**Keywords:** stress granules, poly(ADP-ribose) glycohydrolase, acute kidney injury, renal tubular epithelial cells, pyroptosis

## Abstract

Acute kidney injury (AKI) is a critical clinical syndrome with limited effective therapies, in which renal tubular epithelial cell (RTEC) pyroptosis mediated by the NLRP3 inflammasome represents an important pathological contributor. Stress granules (SGs), dynamic membrane-less condensates, enable cells to adapt to various stress conditions, yet their role and regulatory mechanism in AKI remain unclear. Here, we identify SG persistence as a protective mechanism against AKI and show that poly(ADP-ribosyl)ation (PARylation)-mediated regulation of SG dynamics alleviates pyroptosis during renal injury. We found that SGs were prominently formed in RTECs from AKI patients, cisplatin-induced AKI mice, and cisplatin-stimulated HK-2 cells. Poly(ADP-ribose) glycohydrolase (PARG), the key enzyme for reversing PARylation, was significantly upregulated in injured renal tissues and cells. Genetic or pharmacological inhibition of PARG delayed SG disassembly, enhanced SG persistence, and mitigated renal injury. Mechanistically, persistent SGs regulated DDX3X availability and reduced DDX3X-NLRP3 inflammasome activation, thereby attenuating RTEC pyroptosis. These findings uncover a novel mechanism of SG-mediated renoprotection and highlight SG dynamics as a potential therapeutic target in AKI.

## 1. Introduction

Acute kidney injury (AKI) is a life-threatening clinical syndrome worldwide, with high morbidity and mortality [1,2,3,4]. Despite extensive advances in understanding the pathophysiology of AKI, curative therapies remain lacking, with clinical management mainly limited to supportive care [5]. A central challenge in AKI is mitigating injury to renal tubular epithelial cells (RTECs), which are the primary cellular targets governing disease progression and the ensuing inflammatory cascade. Among the various forms of programmed cell death, pyroptosis has emerged as a key driver of tubular injury [6,7]. This highly inflammatory form of cell death is mediated by activation of the NOD-like receptor protein 3 (NLRP3) inflammasome, leading to caspase-1 activation, Gasdermin-D (GSDMD) cleavage, and the release of inflammatory cytokines such as IL-1β and IL-18. Although the downstream mechanisms of pyroptosis are well characterized, the upstream regulatory pathways determining inflammasome activation in AKI remain poorly understood.

Stress granules (SGs) are dynamic, membrane-less condensates composed of mRNAs, translation initiation factors, 40S ribosomal subunits, and various RNA-binding proteins [8,9,10]. They assemble in the cytoplasm in response to diverse stressors, including hypoxia, oxidative stress, and toxins [11,12,13]. Ras-GAP SH3 domain-binding protein 1 (G3BP1) is a core SG component and plays an essential role in SG assembly. Therefore, G3BP1 is widely used as a representative marker for monitoring SG in response to cellular stress [14]. As a critical adaptive cellular defense mechanism, SGs protect essential mRNAs and functional proteins from abnormal degradation or ectopic activation. Upon stress resolution, SGs are able to rapidly disassemble and release sequestered components, thereby promoting cell survival and repair [15]. A recent study demonstrated that SGs selectively recruit and shelter m^6^A-methylated mRNAs in stressed cells, preventing their aberrant degradation and enhancing cellular resilience [16]. Beyond their classical role in RNA metabolism and translational control, increasing evidence suggests that SGs can regulate cellular signaling by selectively recruiting specific proteins. While SG dysfunction has been implicated in cancer, neurodegeneration, and infectious diseases [17,18,19,20,21], its role in renal injury, particularly in regulating pyroptosis-induced inflammatory signaling pathways, remains unclear.

The dynamic assembly and disassembly of SGs are regulated by post-translational modifications (PTMs) [15,22,23,24,25]. Among these, poly(ADP-ribosyl)ation (PARylation), catalyzed by poly(ADP-ribose) polymerases (PARPs) and reversed by poly(ADP-ribose) glycohydrolase (PARG), is known as a key regulator of SG dynamics [26,27,28,29,30,31]. Previous studies have shown that PARP contributes to SG formation through interactions between PAR and SG-associated proteins [32,33]. However, whether PARG-mediated regulation of SG dynamics has functional consequences in renal injury remains unknown. Since PARP inhibition may influence DNA repair pathways that are important during AKI, we focused on PARG as a potential strategy to modulate SG dynamics [34]. We therefore sought to determine whether PARG-dependent regulation of PARylation controls SG persistence in RTECs and whether this process influences downstream inflammatory signaling, particularly NLRP3 inflammasome-mediated pyroptosis.

DEAD-box helicase 3 X-linked (DDX3X), a multifunctional RNA helicase involved in mRNA metabolism and cellular stress responses, has been implicated in regulating NLRP3 inflammasome activation and pyroptosis in immune cells. Notably, DDX3X is localized to SGs and participates in their assembly in alveolar macrophages, suggesting a potential connection between SG dynamics and inflammasome regulation [35]. However, whether SGs regulate DDX3X availability and thereby influence inflammasome activation during kidney injury remains unknown. The expression pattern and biological function of DDX3X in the kidney and its role in NLRP3-mediated pyroptosis during AKI have not been fully characterized.

In this study, we identify SGs as active regulators of inflammatory signaling in AKI. We show that PARG-dependent control of SG dynamics sustains their protective function, and further demonstrate that this effect is mediated through sequestration of DDX3X, thereby limiting NLRP3 inflammasome activation and pyroptosis. Together, these findings define a previously unrecognized regulatory axis linking SGs to inflammasome signaling and suggest a potential therapeutic strategy for AKI.

## 2. Results

### 2.1. SGs Are Induced in Proximal Tubule Epithelial Cells During AKI

**Figure 1:**
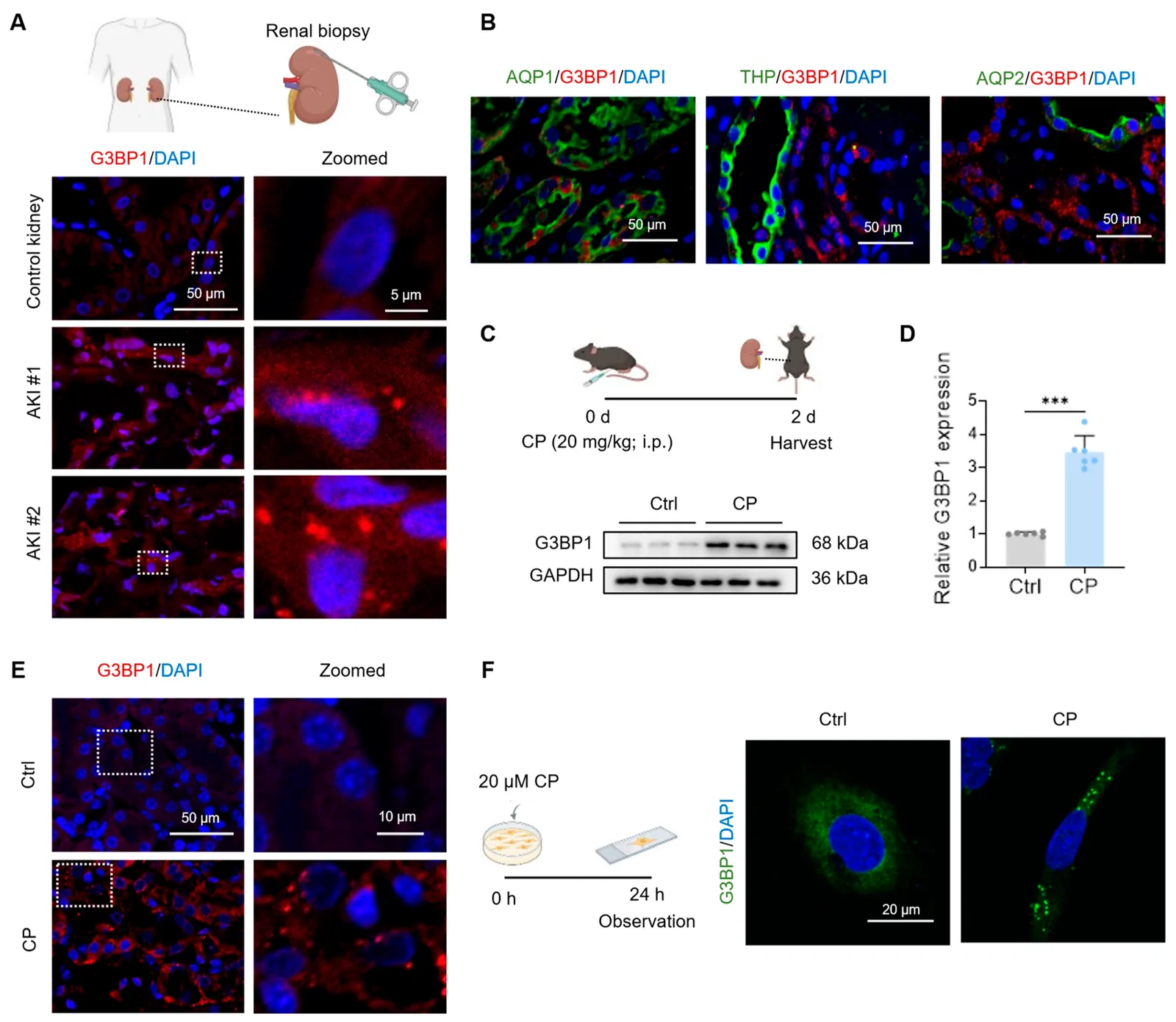
SGs are induced in proximal tubule epithelial cells during AKI. (**A**) Immunofluorescence staining of G3BP1 (red) in renal biopsy specimens from AKI patients and control kidneys from donors, with zoomed views of boxed areas. Scale bars: 50 μm (left panels), 5 μm (right zoomed panels). (**B**) Immunofluorescence co-localization of G3BP1 (red) with the proximal tubular marker AQP1 (green), the distal tubular marker THP (green), and the collecting duct marker AQP2 (green) in AKI renal tissues. Scale bar: 50 μm. (**C**,**D**) Representative Western blot images and quantification of G3BP1 expression in renal tissues from mice. *n* = 6 biological replicates per group. *** *p* < 0.001 vs. Ctrl. (**E**) Immunofluorescence staining of G3BP1 (red) in renal tissues from control and cisplatin-treated mice, with zoomed views of boxed areas. Scale bars: 50 μm (left panels), 10 μm (right zoomed panels). (**F**) Immunofluorescence images of G3BP1 (green) in HK-2 cells. Scale bar: 20 μm. Data were analyzed by Student’s *t*-tests (**D**).

To determine whether SGs are induced during renal injury, we first examined SG formation in human kidney samples from patients with AKI. Immunofluorescence staining revealed abundant G3BP1-positive cytoplasmic puncta in renal tissues from AKI patients, whereas such puncta were rarely observed in control kidney tissues from donors (Figure 1A). Quantification of biopsy specimens further supported the differential presence of G3BP1-positive structures between AKI and control kidneys (Figure S1). Further immunofluorescence co-localization analysis demonstrated that these G3BP1-positive puncta were predominantly localized in AQP1-positive proximal tubular epithelial cells (Figure 1B), indicating that proximal tubular epithelial cells are a major site of SG formation during AKI.

We next established a cisplatin-induced AKI model to examine SG formation. Consistent with the findings in human kidney samples, cisplatin-induced AKI mice exhibited marked upregulation of G3BP1 expression and increased formation of G3BP1-positive puncta in renal tissues (Figure 1C–E). Similar observations were obtained in cisplatin-treated HK-2 cells (Figure 1F). To further verify that G3BP1-positive puncta represented SGs, we performed immunofluorescence co-staining of G3BP1 with another well-established SG marker, TIA-1, in HK-2 cells. G3BP1-positive puncta showed substantial overlap with TIA-1 after cisplatin stimulation, supporting the identification of these structures as SGs (Figure S2). These findings confirm that SG represents a conserved cellular response to renal injury across human, in vivo, and in vitro systems.

### 2.2. PARG Knockdown Delays the Disassembly of SGs

**Figure 2:**
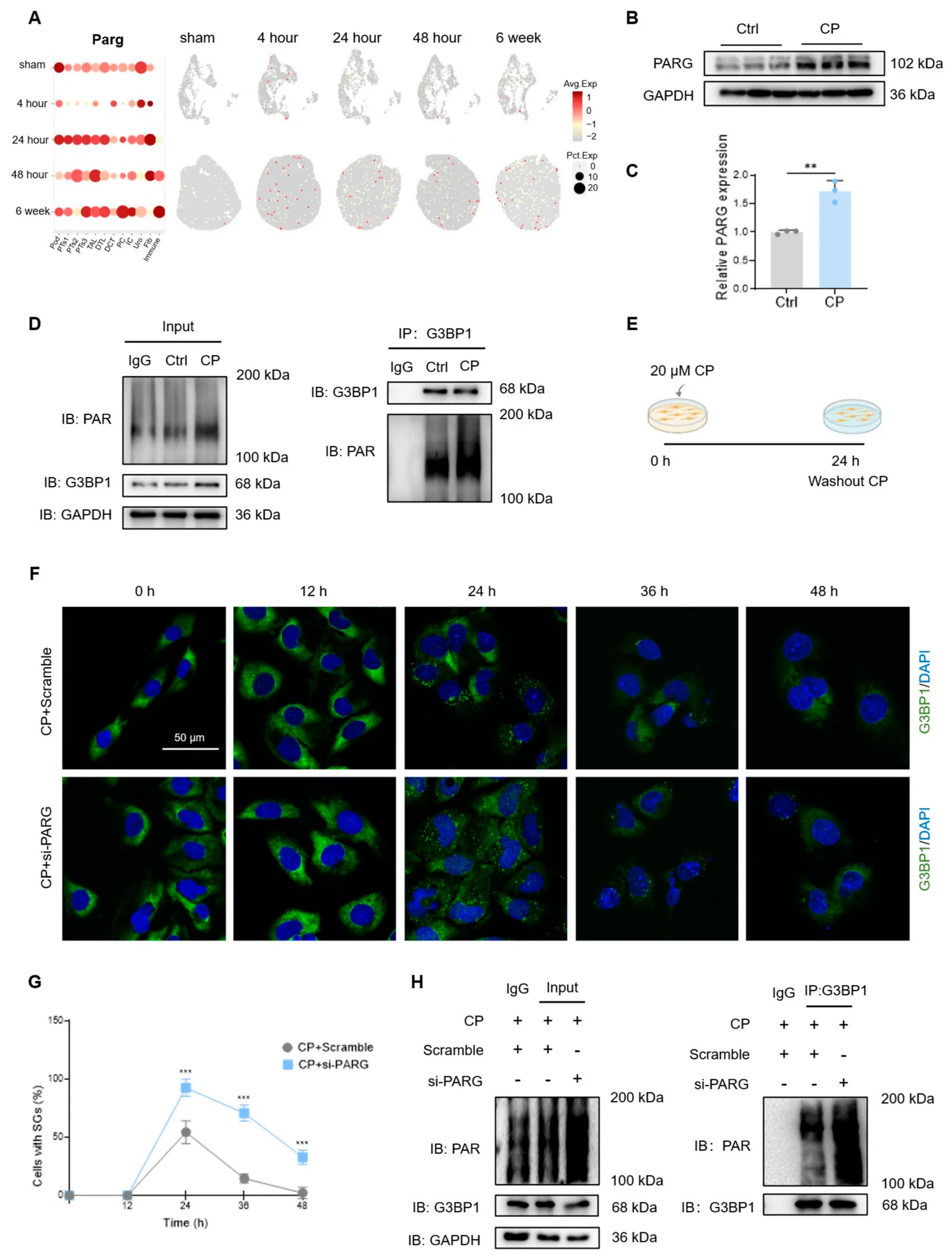
PARG knockdown delays the disassembly of SGs. (**A**) Single-cell transcriptomic analysis of Parg expression in mouse kidneys at different stages of AKI. (**B**,**C**) Representative Western blot images and quantification of PARG expression in HK-2 cells. *n* = 3 biological replicates per group. ** *p* < 0.01 vs. Ctrl. (**D**) Co-IP assay showing the interaction and PARylation level of G3BP1-containing complexes in HK-2 cells. (**E**) Schematic diagram of the SG disassembly assay: HK-2 cells were treated with 20 μM cisplatin for 24 h, followed by thorough cisplatin washout to initiate SG disassembly. (**F**,**G**) Immunofluorescence staining of G3BP1 (green) in cisplatin-treated HK-2 cells after PARG knockdown. Quantification of the percentage of cells with SGs at each time point. Scale bar: 50 μm. *n* = 5 biological replicates per group. *** *p* < 0.001 vs. CP+Scramble. (**H**) Co-IP assay confirming the effect of PARG knockdown on G3BP1-containing complexes PARylation in cisplatin-stimulated HK-2 cells. Data were analyzed by Student’s *t*-tests (**C**) or two-way analysis of variance (ANOVA) with Tukey’s multiple comparisons (**G**).

To investigate whether PARG-mediated PAR metabolism contributes to the regulation of SG dynamics in RTECs, we reanalyzed single-cell transcriptomic datasets from mouse AKI models at different pathological stages (http://humphreyslab.com/SingleCell, accessed on 1 March 2026). Bioinformatic analysis revealed that Parg was upregulated during renal injury progression (Figure 2A). Consistent with the in vivo observations, Western blotting demonstrated markedly increased PARG expression in cisplatin-stimulated HK-2 cells (Figure 2B,C). We next examined G3BP1 PARylation in cisplatin-treated HK-2 cells. Immunoprecipitation (Co-IP) analysis showed that G3BP1-containing complexes underwent PARylation under cisplatin-induced stress (Figure 2D).

To clarify the effect of PARG on renal SG dynamics, HK-2 cells were transfected with siRNA targeting PARG to silence endogenous PARG expression. Cisplatin was then applied to trigger SG formation. After 24 h, cisplatin was thoroughly washed out to initiate SG disassembly (Figure 2E). Immunofluorescence analysis showed that SG formation peaked at 24 h following cisplatin treatment. After cisplatin withdrawal, the disassembly of SGs was significantly delayed in PARG-deficient HK-2 cells (Figure 2F,G). Moreover, PARG knockdown further increased the PARylation level of G3BP1-containing complexes under cisplatin stimulation (Figure 2H). Together, these findings demonstrate that PARG knockdown delays the disassembly of SGs during renal tubular stress.

### 2.3. Delayed SG Disassembly Protects Kidneys in AKI Mice

**Figure 3:**
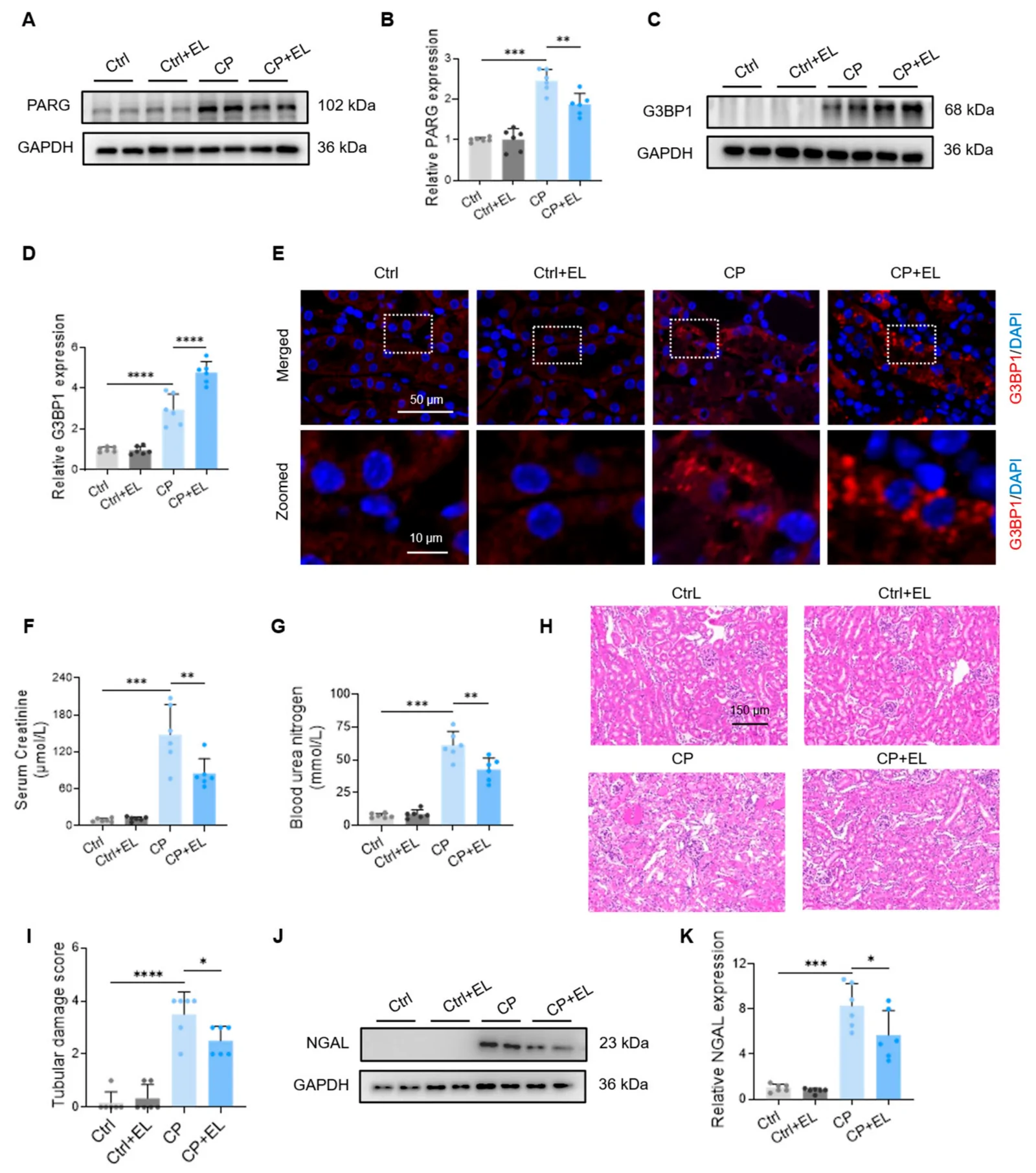
Delayed SG disassembly protects kidneys in AKI mice. (**A**,**B**) Representative Western blot images and quantification of PARG expression in renal tissues. *n* = 6 biological replicates per group. ** *p* < 0.01 vs. CP+EL, *** *p* < 0.001 vs. Ctrl. (**C**,**D**) Representative Western blot images and quantification of G3BP1 expression in renal tissues. *n* = 6 biological replicates per group. **** *p* < 0.0001 vs. Ctrl/CP+EL. (**E**) Immunofluorescence staining of G3BP1 (red) in renal tissues, with zoomed views of boxed areas. Scale bars: 50 μm (upper panels), 10 μm (lower zoomed panels). (**F**) Serum creatinine levels in mice. *n* = 6 biological replicates per group. ** *p* < 0.01 vs. CP+EL, *** *p* < 0.001 vs. Ctrl. (**G**) Blood urea nitrogen levels in mice. *n* = 6 biological replicates per group. ** *p* < 0.01 vs. CP+EL, *** *p* < 0.001 vs. Ctrl. (**H**) HE staining of renal sections. Scale bar: 150 μm. (**I**) Quantification of tubular damage score in renal tissues. *n* = 6 biological replicates per group. * *p* < 0.05 vs. CP+EL, **** *p* < 0.0001 vs. Ctrl. (**J**,**K**) Representative Western blot images and quantification of NGAL expression in renal tissues. *n* = 6 biological replicates per group. * *p* < 0.05 vs. CP+EL, *** *p* < 0.001 vs. Ctrl. Data were analyzed by one-way ANOVA with Tukey’s multiple comparisons (**B**,**D**,**F**,**G**,**I**,**K**).

To further explore the functional significance of delayed SG disassembly in renal injury, we pharmacologically inhibited PARG by intraperitoneal injection of EL in cisplatin-induced AKI mice (Figure 3A,B). Western blotting showed that G3BP1 expression was significantly increased in the renal tissues of EL-treated AKI mice compared with cisplatin-treated mice (Figure 3C,D). Consistently, immunofluorescence analysis revealed enlarged SGs in the kidneys of mice receiving EL intervention (Figure 3E), indicating that pharmacological inhibition of PARG promotes SG persistence during renal injury.

We next evaluated the protective effects of delayed SG disassembly in vivo. The results showed that EL significantly reduced serum creatinine (Figure 3F) and blood urea nitrogen levels (Figure 3G) in AKI mice. Hematoxylin and eosin (HE) staining showed that EL alleviated typical renal tubular injuries, including tubular epithelial cell shedding, luminal dilation, and cast formation (Figure 3H). Meanwhile, EL treatment significantly reduced tubular injury scores in AKI mice (Figure 3I). Furthermore, Western blotting confirmed that the renal expression of NGAL, a sensitive tubular injury biomarker, was significantly decreased in EL-treated AKI mice (Figure 3J,K).

These results indicate that pharmacological inhibition of PARG-mediated SG disassembly preserves SG persistence and attenuates renal injury in cisplatin-induced AKI mice.

### 2.4. Delayed SG Disassembly Protects HK-2 Cells Against Cisplatin-Induced Injury

**Figure 4:**
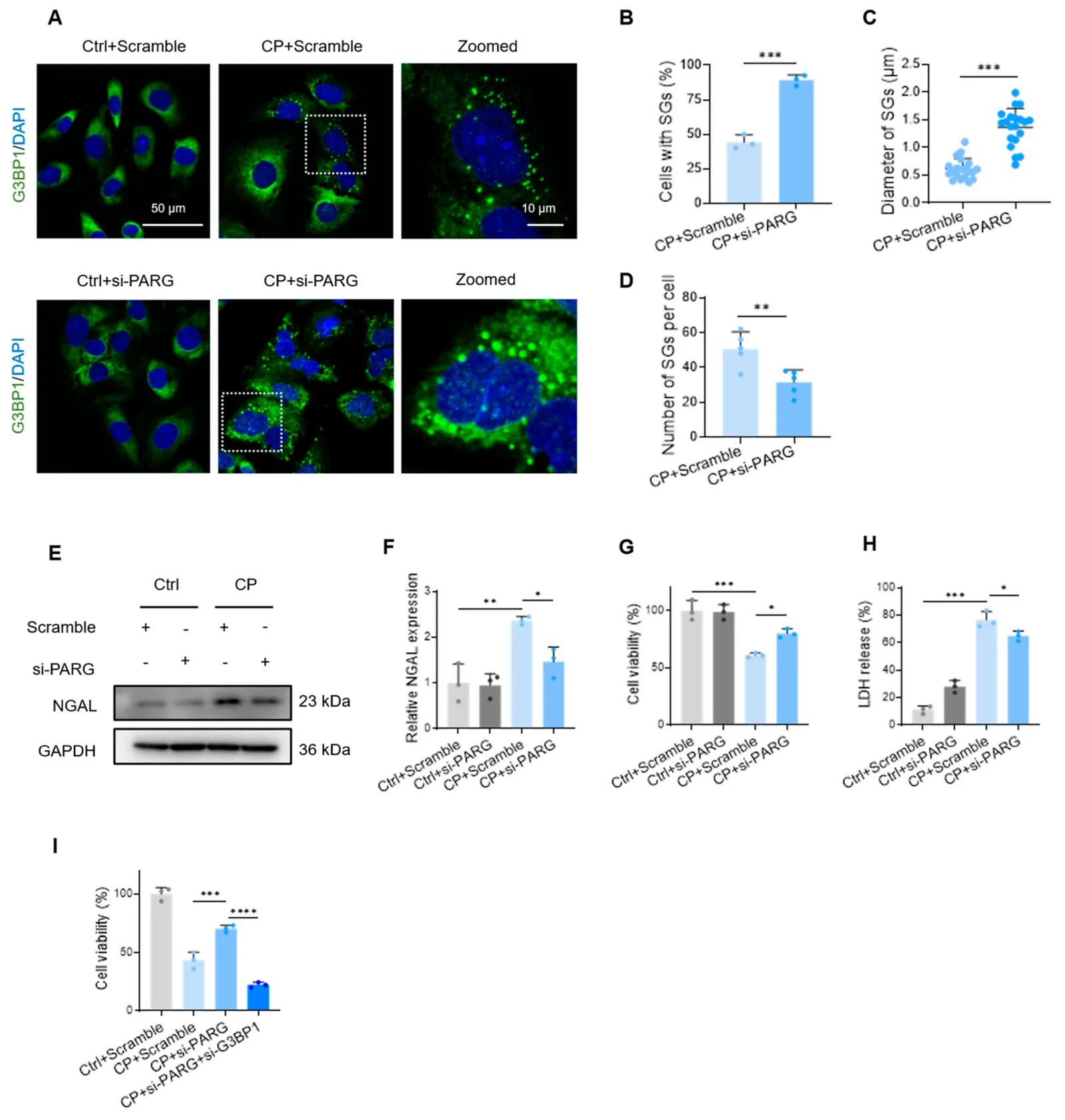
Delayed SG disassembly protects HK-2 cells against cisplatin-induced injury. (**A**) Immunofluorescence staining of G3BP1 (green) in HK-2 cells after PARG knockdown, with zoomed views of boxed areas. Scale bars: 50 μm (left panels), 10 μm (right zoomed panels). (**B**) Quantification of the percentage of cells with SGs. *n* = 3 independent experiments. *** *p* < 0.001 vs. CP+Scramble. (**C**) Quantification of the average diameter of individual SGs. *n* = 3 independent experiments, 6 randomly selected SGs analyzed per group. *** *p* < 0.001 vs. CP+Scramble. (**D**) Quantification of the number of SGs per cell. *n* = 5 independent experiments. ** *p* < 0.01 vs. CP+Scramble. (**E**,**F**) Representative Western blot images and quantification of NGAL expression in HK-2 cells. *n* = 3 biological replicates per group. * *p* < 0.05 vs. CP+si-PARG, ** *p* < 0.01 vs. Ctrl+Scramble. (**G**) Cell viability assays in HK-2 cells. *n* = 3 biological replicates per group. * *p* < 0.05 vs. CP+si-PARG, *** *p* < 0.001 vs. Ctrl+Scramble. (**H**) LDH release assays in HK-2 cells. *n* = 3 biological replicates per group. * *p* < 0.05 vs. CP+si-PARG, *** *p* < 0.001 vs. Ctrl+Scramble. (**I**) Cell viability assays in HK-2 cells transfected with si-PARG and si-G3BP1. *n* = 3 biological replicates per group. *** *p* < 0.001 vs. CP+Scramble, **** *p* < 0.0001 vs. CP+ si-PARG+ si-G3BP1. Data were analyzed by Student’s *t*-tests (**B**–**D**) or one-way ANOVA with Tukey’s multiple comparisons (**F**–**I**).

For in vitro validation, we delayed SG disassembly by siRNA-mediated PARG knockdown. Following transfection with si-PARG and cisplatin stimulation, both the proportion of SG-positive cells and the average diameter of individual SGs were increased, while the number of SGs per cell was reduced in HK-2 cells (Figure 4A–D). Consistent with our previous findings, PARG knockdown significantly suppressed NGAL expression in cisplatin-treated HK-2 cells (Figure 4E,F). Functionally, PARG knockdown restored cell viability impaired by cisplatin (Figure 4G) and attenuated cisplatin-triggered lactate dehydrogenase (LDH) release (Figure 4H).

To further determine whether SG contributes to the protective effect associated with PARG inhibition, we performed loss-of-function experiments targeting G3BP1. HK-2 cells were transfected with si-PARG alone or co-transfected with si-PARG and si-G3BP1 before cisplatin stimulation. Inhibition of G3BP1-mediated SG formation markedly reduced the protective effect of PARG depletion. Compared with cells treated with si-PARG alone, cells receiving combined si-PARG and si-G3BP1 showed decreased cell viability following cisplatin exposure (Figure 4I).

### 2.5. Delayed SG Disassembly Inhibits Pyroptosis in Cisplatin-Stimulated HK-2 Cells

**Figure 5:**
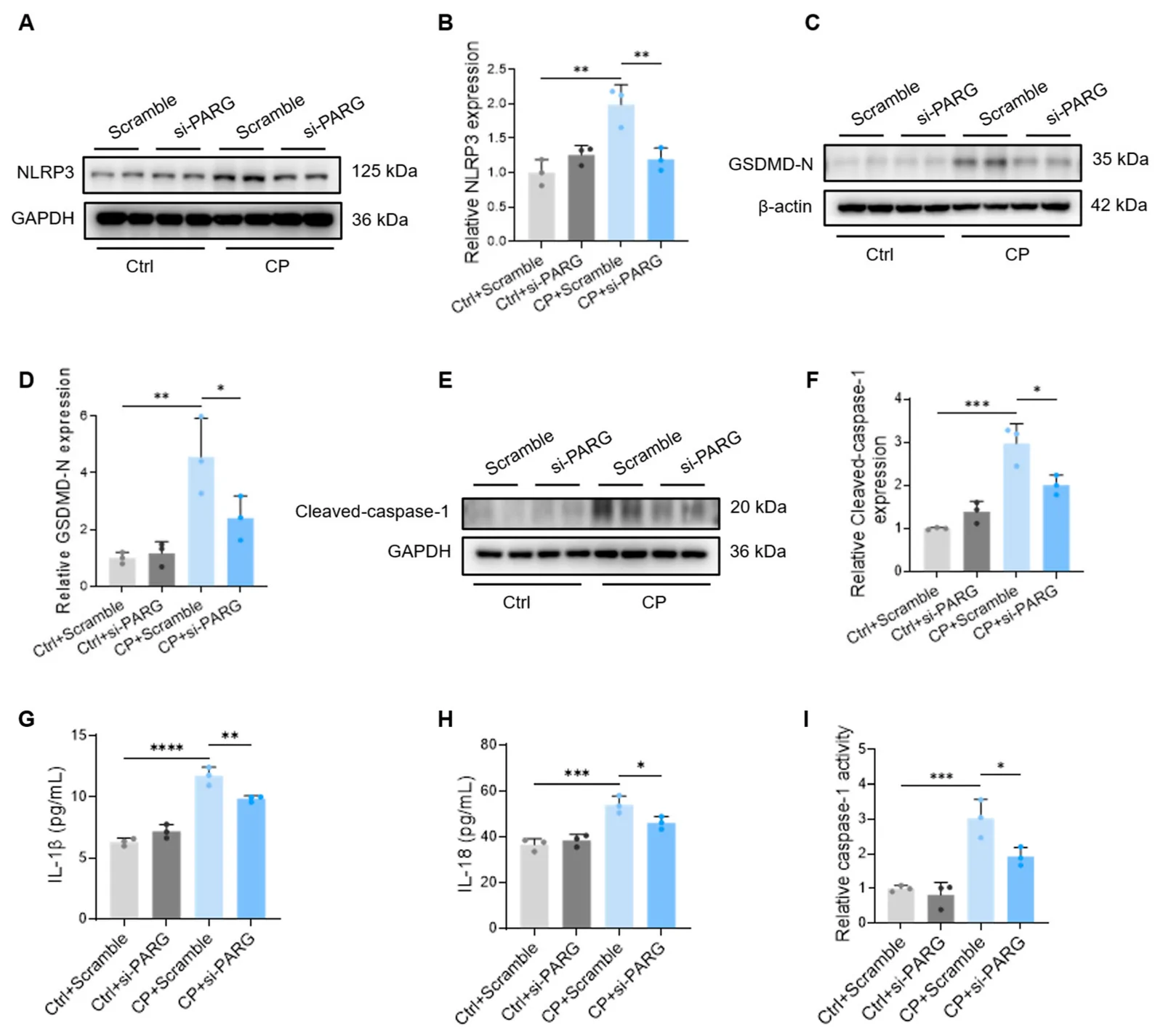
Delayed SG disassembly inhibits pyroptosis in cisplatin-stimulated HK-2 cells. (**A**,**B**) Representative Western blot images and quantification of NLRP3 expression in HK-2 cells. *n* = 3 biological replicates per group. ** *p* < 0.01 vs. Ctrl+Scramble/CP+si-PARG. (**C**,**D**) Representative Western blot images and quantification of GSDMD-N expression in HK-2 cells. *n* = 3 biological replicates per group. * *p* < 0.05 vs. CP+si-PARG, ** *p* < 0.01 vs. Ctrl+Scramble. (**E**,**F**) Representative Western blot images and quantification of cleaved-caspase-1 expression in HK-2 cells. *n* = 3 biological replicates per group. * *p* < 0.05 vs. CP+si-PARG, *** *p* < 0.001 vs. Ctrl+Scramble. (**G**) ELISA of IL-1β in the culture supernatant of HK-2 cells. *n* = 3 biological replicates per group. ** *p* < 0.01 vs. CP+si-PARG, **** *p* < 0.0001 vs. Ctrl+Scramble. (**H**) ELISA of IL-18 in the culture supernatant of HK-2 cells. *n* = 3 biological replicates per group. * *p* < 0.05 vs. CP+si-PARG, *** *p* < 0.001 vs. Ctrl+Scramble. (**I**) Caspase-1 activity in HK-2 cells transfected with si-PARG. *n* = 3 biological replicates per group. * *p* < 0.05 vs. CP+si-PARG, *** *p* < 0.001 vs. Ctrl+Scramble. Data were analyzed by one-way ANOVA with Tukey’s multiple comparisons (**B**,**D**,**F**–**I**).

Pyroptosis constitutes a critical pattern of tubular epithelial cell death during AKI (Figures S3 and S4). To determine whether SGs modulate pyroptosis, we delayed SG disassembly in HK-2 cells by silencing PARG. Western blot analysis showed that PARG knockdown significantly decreased the expression of pyroptosis-associated proteins, including NLRP3 (Figure 5A,B), GSDMD-N (Figure 5C,D), and cleaved-caspase-1 (Figure 5E,F), following cisplatin stimulation. Meanwhile, enzyme-linked immunosorbent assay (ELISA) analysis demonstrated that PARG depletion reduced the secretion of inflammatory cytokines IL-1β (Figure 5G) and IL-18 (Figure 5H). Consistently, caspase-1 activity was also reduced after PARG silencing (Figure 5I).

These findings indicate that PARG inhibition-mediated SG persistence attenuates pyroptosis in cisplatin-stressed HK-2 cells.

### 2.6. Delayed SG Disassembly Inhibits Renal Pyroptosis in AKI Mice

**Figure 6:**
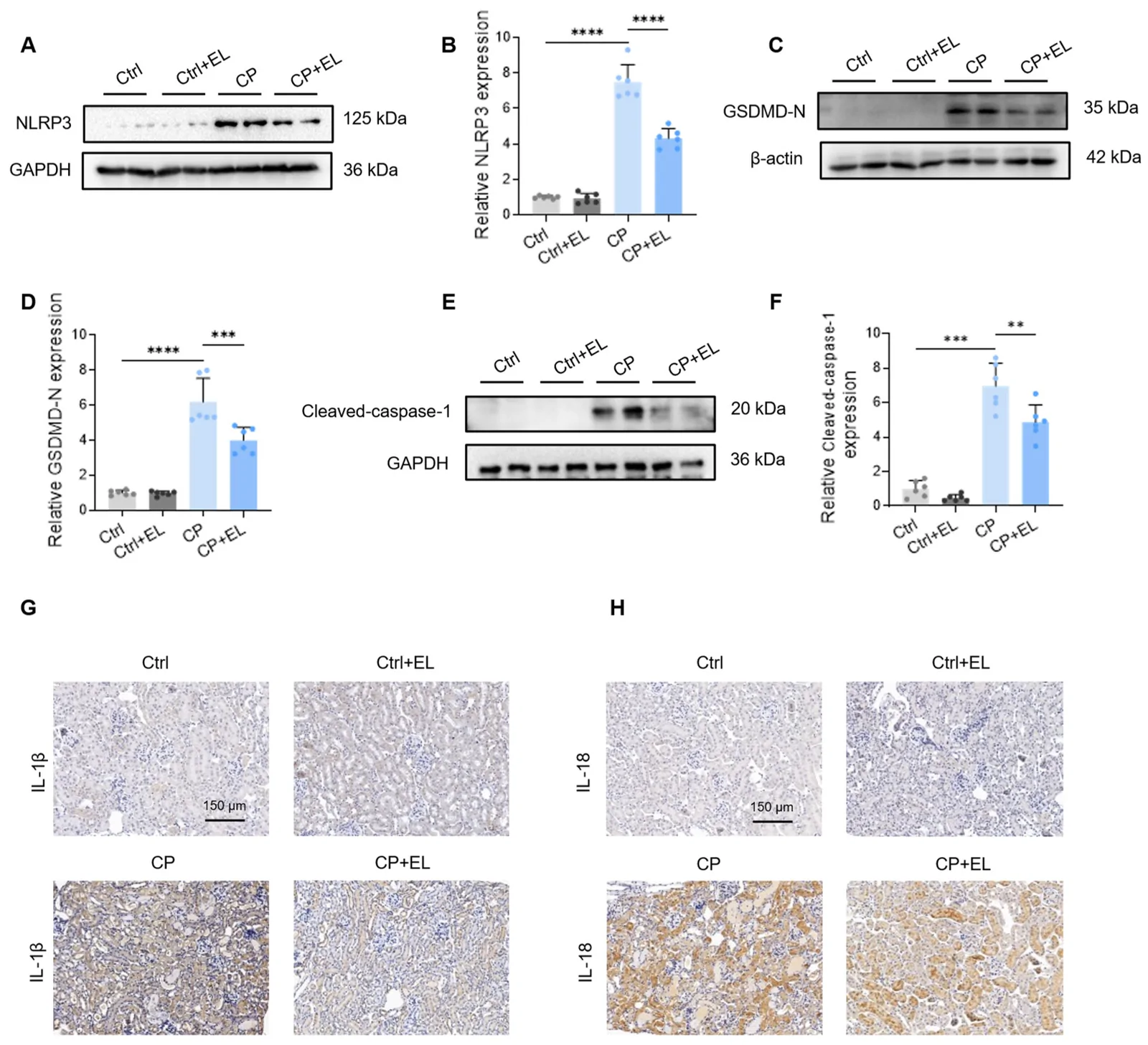
Delayed SG disassembly inhibits renal pyroptosis in AKI mice. (**A**,**B**) Representative Western blot images and quantification of NLRP3 expression in renal tissues. *n* = 6 biological replicates per group. **** *p* < 0.0001 vs. Ctrl/CP+EL. (**C**,**D**) Representative Western blot images and quantification of GSDMD-N expression in renal tissues. *n* = 6 biological replicates per group. *** *p* < 0.001 vs. CP+EL, **** *p* < 0.0001 vs. Ctrl. (**E**,**F**) Representative Western blot images and quantification of cleaved-caspase-1 expression in renal tissues. *n* = 6 biological replicates per group. ** *p* < 0.01 vs. CP+EL, *** *p* < 0.001 vs. Ctrl. (**G**) IHC staining of IL-1β of renal sections. Scale bar: 150 μm. (**H**) IHC staining of IL-18 in renal sections. Scale bar: 150 μm. Data were analyzed by one-way ANOVA with Tukey’s multiple comparisons (**B**,**D**,**F**).

Consistent with the in vitro findings, the pharmacologically delayed SG disassembly by EL treatment reduced renal expression of NLRP3 (Figure 6A,B), GSDMD-N (Figure 6C,D), and cleaved caspase-1 (Figure 6E,F). Immunohistochemical (IHC) staining further demonstrated decreased renal expression of IL-1β (Figure 6G) and IL-18 following EL treatment (Figure 6H).

These results demonstrate that delayed SG disassembly effectively suppresses pyroptosis during cisplatin-induced AKI in mice.

### 2.7. SG Persistence Attenuates Pyroptosis by Regulating DDX3X-NLRP3 Signaling

**Figure 7:**
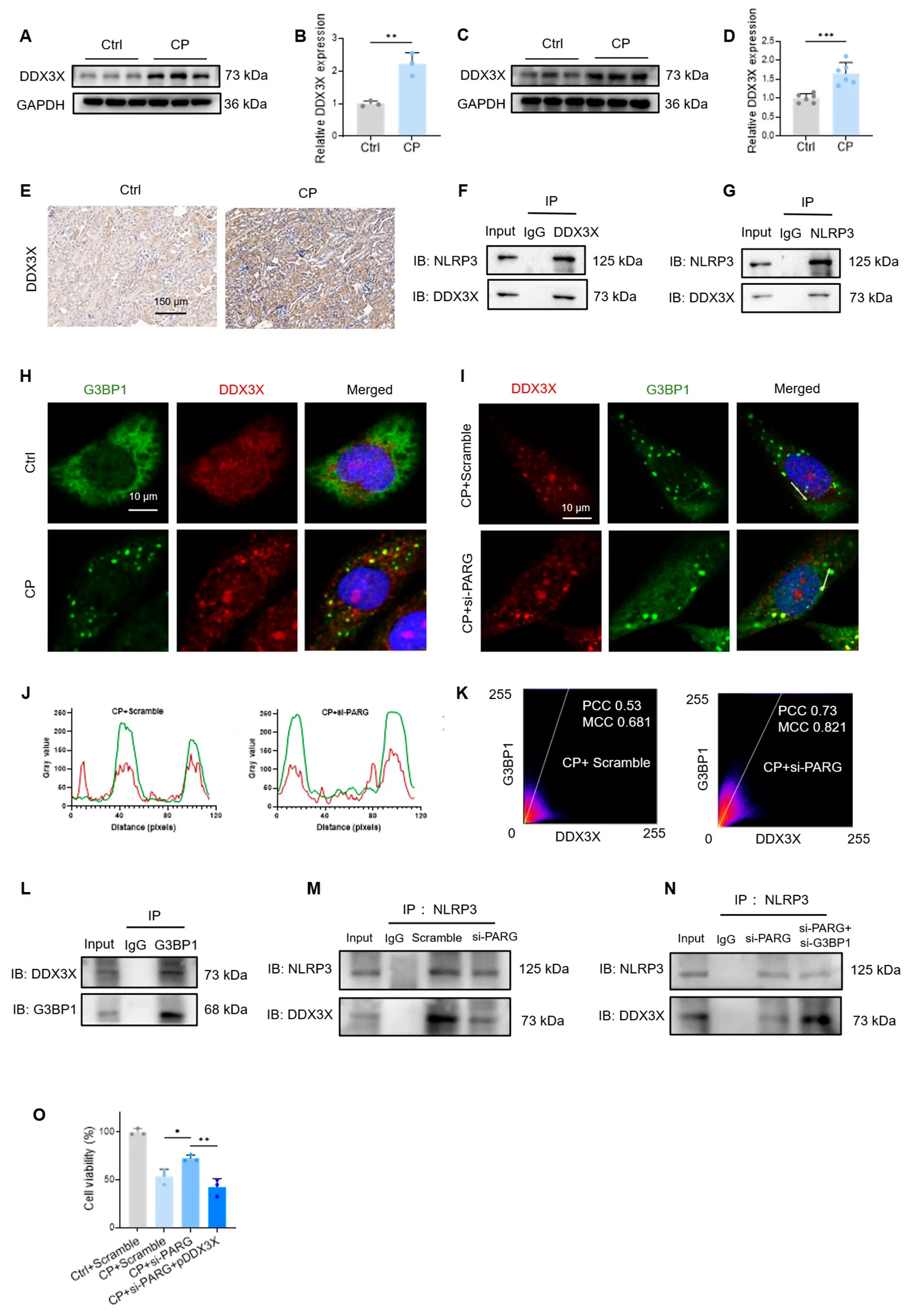
SG persistence attenuates pyroptosis by regulating DDX3X-NLRP3 signaling. (**A**,**B**) Representative Western blot images and quantification of DDX3X expression in HK-2 cells. *n* = 3 biological replicates per group. ** *p* < 0.01 vs. Ctrl. (**C**,**D**) Representative Western blot images and quantification of DDX3X expression in renal tissues from different groups of mice. *n* = 6 biological replicates per group. *** *p* < 0.001 vs. Ctrl. (**E**) IHC staining of DDX3X in renal sections. Scale bar: 150 μm. (**F**,**G**) Co-IP assays demonstrating the interaction between DDX3X and NLRP3 in cisplatin-stimulated HK-2 cells. (**H**) Immunofluorescence co-localization of G3BP1 (green) and DDX3X (red) in HK-2 cells. Scale bar: 10 μm. (**I**,**J**) Immunofluorescence staining and line-scan analysis of DDX3X (red) and G3BP1 (green) in cisplatin-treated HK-2 cells after PARG knockdown. Scale bar: 10 μm. (**K**) Pearson’s correlation coefficient (PCC) and Manders’ colocalization coefficient (MCC) analysis of DDX3X and G3BP1 co-localization. (**L**) Co-IP assays demonstrating the interaction between DDX3X and G3BP1-containing complexes in cisplatin-stimulated HK-2 cells. (**M**) Co-IP assay confirming the influence of PARG knockdown on the interaction between DDX3X and NLRP3 in cisplatin-stimulated HK-2 cells. (**N**) Co-IP assay confirming the influence of G3BP1 knockdown on the interaction between DDX3X and NLRP3 in cisplatin-stimulated PARG-deficient cells. (**O**) Cell viability assays in cisplatin-stimulated HK-2 cells transfected with si-PARG and pDDX3X. *n* = 3 biological replicates per group. * *p* < 0.05 vs. CP+Scramble, ** *p* < 0.01 vs. CP+ si-PARG+ pDDX3X. Data were analyzed by Student’s *t*-tests (**B**,**D**) or one-way ANOVA with Tukey’s multiple comparisons (**O**).

Given that SGs do not directly interact with or regulate NLRP3 (Figure S5), we hypothesized that intermediate molecules might bridge SGs and NLRP3 inflammasome-mediated pyroptosis. We focused on DDX3X, a known activator of the NLRP3 inflammasome in macrophages, whose expression and function in the kidney remain poorly defined. We first detected DDX3X expression in AKI models. In vitro, cisplatin treatment significantly upregulated DDX3X expression in HK-2 cells (Figure 7A,B). This upregulation was recapitulated in vivo, as DDX3X expression was markedly increased in renal tissues from cisplatin-induced AKI mice (Figure 7C,D). IHC staining further confirmed the increased DDX3X expression in renal tissues of cisplatin-induced AKI mice (Figure 7E), consistent with our Western blot results. Co-IP assays further demonstrated a direct interaction between DDX3X and NLRP3 in cisplatin-stimulated HK-2 cells (Figure 7F,G). Furthermore, to verify the regulatory role of DDX3X in NLRP3 inflammasome-mediated pyroptosis, we performed in vitro and in vivo experiments. In HK-2 cells, transfection with pDDX3X significantly enhanced cell pyroptosis (Figure S6), whereas treatment with the DDX3X inhibitor RK-33 markedly attenuated this effect (Figure S7). In vivo, renal overexpression of DDX3X via AAV injection aggravated renal pyroptosis (Figure S8). Collectively, these results confirmed that DDX3X functions as a positive regulator of the NLRP3 inflammasome in the kidney.

Notably, SGs did not alter the total expression of DDX3X (Figure S9). We thus examined the subcellular distribution of DDX3X relative to SGs by immunofluorescence co-staining. Obvious co-localization between DDX3X and G3BP1 was observed in cisplatin-treated HK-2 cells (Figure 7H). Further analysis showed that PARG knockdown enhanced the co-localization of DDX3X with SGs (Figure 7I,J), accompanied by an increased Manders’ co-localization coefficient (Figure 7K). In addition, Co-IP assays confirmed that DDX3X interacts with G3BP1-containing SG complexes (Figure 7L). These results indicated that SG may regulate the intracellular availability of DDX3X during injury.

We next examined whether PARG-dependent regulation of SG persistence affects DDX3X-mediated inflammasome activation. Co-IP analysis showed that PARG knockdown reduced the interaction between DDX3X and NLRP3 (Figure 7M), indicating that enhanced SG persistence limits the association of DDX3X with NLRP3. Importantly, inhibition of SG formation by simultaneous G3BP1 knockdown restored the interaction between DDX3X and NLRP3 in PARG-deficient cells (Figure 7N).

To further determine the functional significance of DDX3X regulation, we performed rescue experiments by overexpressing DDX3X in PARG-deficient HK-2 cells. DDX3X overexpression weakened the protective effects induced by PARG knockdown, as demonstrated by reduced cell viability (Figure 7O).

Together, these findings indicate that SG persistence contributes to suppression of pyroptosis through regulation of DDX3X-NLRP3 signaling. DDX3X sequestration represents an important mechanism linking SG dynamics with inflammasome regulation during AKI.

### 2.8. Delayed SG Disassembly Attenuates Pyroptosis Under AAV-Induced DDX3X Overexpression Conditions in AKI Mice

**Figure 8:**
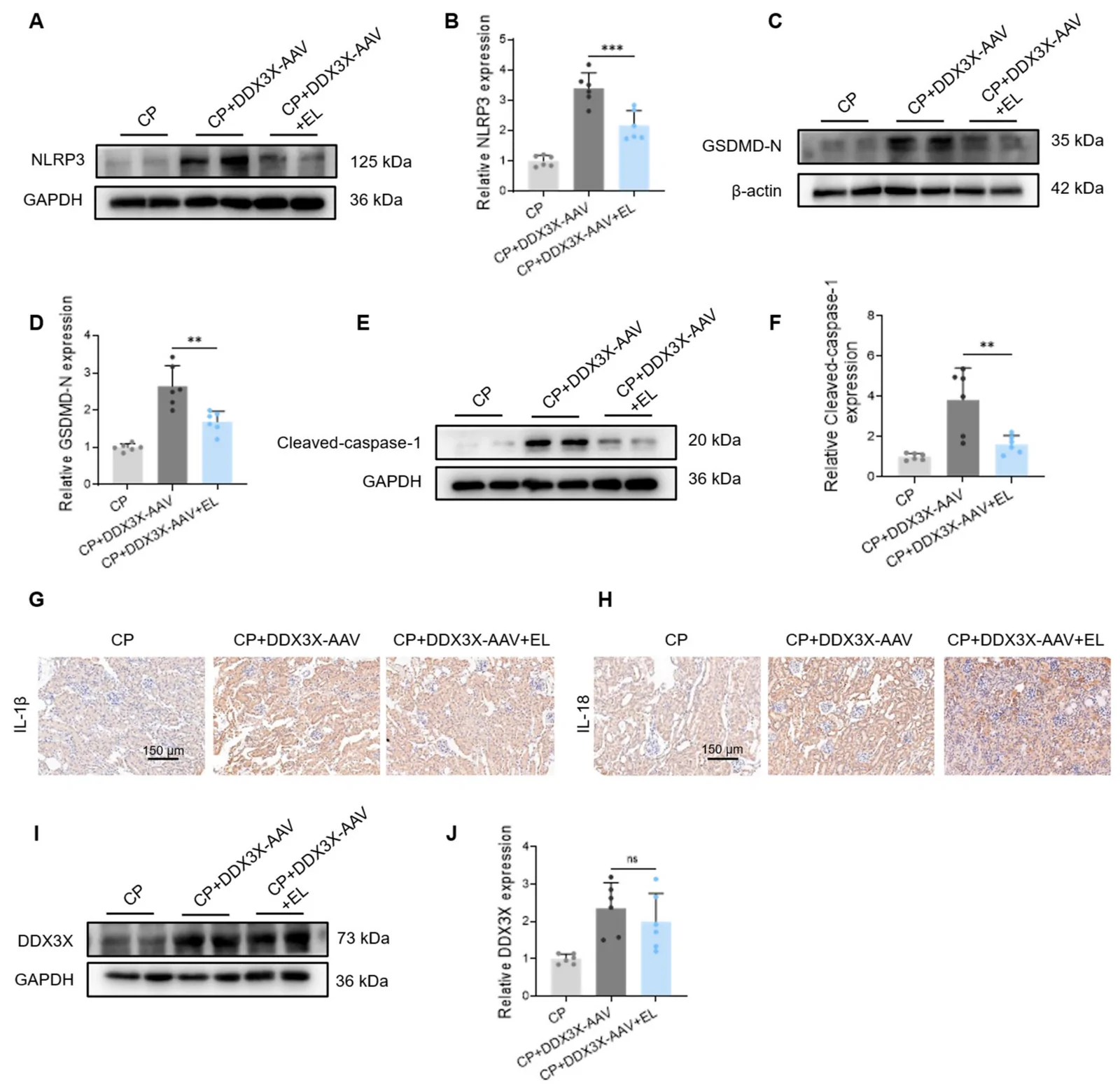
Delayed SG disassembly attenuates pyroptosis under AAV-induced DDX3X overexpression conditions in AKI mice. (**A**,**B**) Representative Western blot images and quantification of NLRP3 expression in renal tissues. *n* = 6 biological replicates per group. *** *p* < 0.001 vs. CP+DDX3X-AAV. (**C**,**D**) Representative Western blot images and quantification of GSDMD-N expression in renal tissues. *n* = 6 biological replicates per group. ** *p* < 0.01 vs. CP+DDX3X-AAV. (**E**,**F**) Representative Western blot images and quantification of cleaved-caspase-1 expression in renal tissues. *n* = 6 biological replicates per group. ** *p* < 0.01 vs. CP+DDX3X-AAV. (**G**) IHC staining of IL-1β in renal sections. Scale bar: 150 μm. (**H**) IHC staining of IL-18 in renal sections. Scale bar: 150 μm. (**I**,**J**) Representative Western blot images and quantification of DDX3X expression in renal tissues. *n* = 6 biological replicates per group. ns, not significant vs. CP+DDX3X-AAV. Data were analyzed by one-way ANOVA with Tukey’s multiple comparisons (**B**,**D**,**F**,**J**).

To further investigate whether modulation of SG dynamics influences pyroptosis under conditions of increased DDX3X expression, we established an AKI mouse model with renal DDX3X overexpression by injecting AAV into the renal cortex. Compared with the Con-AAV group, the DDX3X-AAV group exhibited significantly increased levels of DDX3X (Figure S8). EL was then administered to pharmacologically delay SG disassembly. Compared with AKI mice receiving DDX3X-AAV, EL administration significantly reduced the expression of pyroptosis-associated proteins, including NLRP3 (Figure 8A,B), GSDMD-N (Figure 8C,D), and cleaved-caspase-1 (Figure 8E,F). In addition, EL treatment decreased the renal expression of IL-1β (Figure 8G) and IL-18 (Figure 8H).

Notably, EL did not significantly alter total DDX3X expression (Figure 8I,J), suggesting that the protective effects associated with delayed SG disassembly may involve regulation of DDX3X availability rather than changes in DDX3X abundance.

These findings suggest that enhanced SG persistence can attenuate pyroptosis under AAV-induced DDX3X overexpression conditions. But future studies incorporating AAV control groups and PARG-deficient mice will further establish the causal relationship.

## 3. Discussion

Acute kidney injury remains a major clinical challenge, and effective strategies to prevent tubular injury are still limited. In this study, we identified SG as an endogenous protective mechanism during AKI. We demonstrated that SGs were induced in RTECs following injury and that PARG-mediated regulation of SG dynamics influenced their persistence. Genetic or pharmacological inhibition of PARG delayed SG disassembly and alleviated renal injury. Mechanistically, persistent SGs regulated DDX3X availability and reduced NLRP3 inflammasome activation, thereby limiting pyroptosis. These findings reveal a previously unrecognized connection between SG dynamics and inflammasome regulation in AKI.

An interesting finding in our study is the preferential formation of SGs in proximal tubular epithelial cells during AKI. The proximal tubule is the most metabolically active segment of the nephron and is responsible for extensive reabsorption, resulting in high energy demands and increased susceptibility to metabolic stress, oxidative stress, and toxic injury [36]. These intrinsic characteristics may contribute to the preferential induction of SGs in proximal tubular cells during AKI. A similar pattern is observed in neurons, which exhibit high energetic demand and SG assembly under stress conditions such as ischemia and toxic insults [37,38], suggesting that SG formation might represent a conserved adaptive response in metabolically active cells. In addition, single-cell transcriptomic analysis suggested that PARG expression was relatively enriched in proximal tubular epithelial cells. Therefore, differential PARG expression may partially contribute to the cell-type specificity of SG. However, additional factors, including differences in stress-sensing pathways and phase-separation regulators, may also participate in determining specific SG formation and require further investigation.

SGs have traditionally been considered adaptive structures that maintain cellular homeostasis by regulating mRNA storage, translation, and RNA metabolism [39,40]. However, increasing evidence indicates that SGs also regulate cellular signaling through selective recruitment and sequestration of proteins. Our findings extend the functional understanding of SGs by demonstrating that SG persistence influences inflammatory signaling during renal injury. Rather than serving merely as markers of cellular stress, SGs act as dynamic regulatory compartments that determine the activity and availability of stress-responsive proteins.

PARylation has emerged as an important regulator of SG dynamics. Previous studies have shown that PARylation contributes to SG assembly through interactions between PAR and SG-associated proteins, such as the heterogeneous nuclear ribonucleoprotein A1 [29]. In this study, we found that PARG inhibition increased G3BP1-containing complexes’ PARylation and prolonged SG persistence during renal stress.

DDX3X has been identified as an important regulator of NLRP3 inflammasome activation. Our study demonstrates that persistent SGs regulate DDX3X-NLRP3 signaling during AKI. We found that PARG inhibition reduced the interaction between DDX3X and NLRP3, whereas inhibition of SG formation restored this interaction. Furthermore, DDX3X overexpression weakened the protective effects associated with PARG inhibition. These findings support that SG-mediated regulation of DDX3X availability contributes to suppression of inflammasome activation. However, DDX3X sequestration constitutes one important mechanism mediating SG-dependent protection, while SGs may regulate additional stress signaling pathways. Yang et al. [16] reported that SGs protect renal tubular cells by regulating mRNA stability through m6A-associated pathways. In contrast, our study identifies an additional function of SGs in regulating inflammatory signaling through protein sequestration. These mechanisms are not mutually exclusive. Instead, SG-mediated RNA regulation and protein sequestration may represent parallel protective responses that collectively determine tubular cell fate during AKI. Further studies are needed to determine whether these pathways interact during renal injury.

Although SGs provide protection during AKI, prolonged SG accumulation may have detrimental consequences. Aberrant SG accumulation has been associated with impaired proteostasis, abnormal phase separation, and accumulation of aggregation-prone proteins in chronic stress conditions [41,42,43,44]. Therefore, the duration and context of SG persistence may determine whether SGs exert beneficial or harmful effects. In AKI, transient enhancement of SG persistence promotes cellular adaptation and survival, whereas chronic or excessive SG accumulation may interfere with normal cellular function. Understanding the timing and intensity of SG dynamics regulation will be important for developing SG-targeted therapeutic strategies.

Several limitations of this study should be acknowledged. First, we found that PARG inhibition increased G3BP1-containing complexes’ PARylation and was associated with prolonged SG persistence. However, the detailed molecular mechanisms by which PAR metabolism regulates SG dynamics remain incompletely understood. Whether other PARylated SG-associated proteins contribute to this process remains unclear. Future studies using PARylation-deficient mutants or genetic models will be required to further define these mechanisms. Second, although EL has been reported to possess PARG inhibitory activity, it is not a highly selective PARG inhibitor and may exert additional biological effects. Therefore, the renal protective effects observed with EL treatment cannot be attributed exclusively to PARG inhibition. In this study, genetic manipulation of PARG in vitro, together with SG-dependent rescue experiments and DDX3X functional validation, provided mechanistic support for the involvement of PARG and SGs in mediating pyroptosis during AKI. Future studies using PARG-deficient mice will be necessary to further validate this therapeutic strategy. Third, the human kidney sample size in this study was limited, and AKI represents a heterogeneous clinical condition with diverse etiologies. Larger clinical cohorts will be required to further validate the relevance of SG regulation in human AKI.

## 4. Materials and Methods

### 4.1. Human Specimens

Human renal biopsy specimens were acquired from Union Hospital, Tongji Medical College, Huazhong University of Science and Technology. AKI specimens were obtained from patients clinically and pathologically diagnosed with AKI via renal biopsy. Control specimens were collected from donor kidneys undergoing transplantation evaluation and included when clinical assessment and pathological examination showed no significant renal abnormalities. Clinical characteristics of human kidney samples are summarized in Supplementary Table S1. Informed consent was obtained from all participants, and this study was approved by the Medical Ethics Committee of Union Hospital, Tongji Medical College, Huazhong University of Science and Technology. All protocols adhered to the principles outlined in the Declaration of Helsinki.

### 4.2. Animal Models

Male C57BL/6 mice (6–8 weeks old, 22–25 g) were purchased from Shulaibao Biotechnology (Wuhan, China) and housed under specific pathogen-free (SPF) conditions with a 12 h light/dark cycle. All animal experiments were approved by the Institutional Animal Care and Use Committee of Huazhong University of Science and Technology.

All mice were randomly divided into different groups using an online randomization tool (Research Randomizer, version 4.0). Anesthesia was induced by intraperitoneal injection of 0.5% pentobarbital sodium (Sigma, St. Louis, MO, USA). To establish cisplatin-induced AKI, mice received a single intraperitoneal injection of cisplatin (HY-17394, MCE, Monmouth Junction, NJ, USA) at 20 mg/kg. For the ethacridine lactate (EL; HY-B2174, MCE, USA) treatment, 5 mg/kg/d EL was administered intraperitoneally for 5 consecutive days, including 3 days before cisplatin injection and 2 days after cisplatin injection.

All animals were sacrificed under anesthesia 2 days after cisplatin administration, and kidney tissues were harvested for subsequent analysis.

### 4.3. Overexpression of DDX3X In Vivo

Adeno-associated virus (AAV) overexpressing DDX3X (DDX3X-AAV) and control AAV (Con-AAV) were purchased from Vigene Biosciences (Jinan, China). The viral titer was 2.92 × 1013 vg/mL. Thirty days before AKI modeling, mice were anesthetized, and the kidneys were exposed via a dorsal incision. A total of 100 μL of viral suspension (diluted to 2.92 × 1010 vg/mL) was injected into the renal cortex at multiple sites using an 8 mm insulin syringe. Successful injection was indicated by the blanching of the renal cortex. The needle was held in place for several seconds before withdrawal to prevent leakage.

### 4.4. Cell Culture and Treatment

The human kidney tubular epithelial cell line, HK-2, was obtained from the Nephrology Laboratory, Union Hospital, Tongji Medical College, Huazhong University of Science and Technology. HK-2 cells were cultured in DMEM/F12 (Gibco, Grand Island, NY, USA) supplemented with 10% fetal bovine serum (FBS; Sartorius, Göttingen, Germany) at 37 °C in a 5% CO_2_ atmosphere. After overnight starvation in DMEM/F12 medium with 0.5% FBS, HK-2 cells were treated with 20 μM cisplatin for 24 h. For RK-33 (HY-100455, MCE, USA) treatment, HK-2 cells were preincubated with 5 μM RK-33 for 24 h before cisplatin stimulation.

For gene silencing, small interfering RNA (siRNA) targeting human PARG (sense: 5′-GGGCGGUGAAGUUAGAUUACATT-3′, antisense: 5′-UAAUCUAACUUCACCGCCCUUTT-3′) was synthesized by AuGCT Biotech (Beijing, China). siRNA targeting human G3BP1 (sense: 5′-AUGAAUCUCCUCAAAGCCUGGTT-3′, antisense: 5′-CCAGGCUUUGAGGAGAUUCAUTT-3′) was synthesized by AuGCT Biotech (China). For overexpression, the DDX3X overexpression plasmid (pDDX3X) was constructed by GeneChem (Shanghai, China). HK-2 cells were transfected with 100 pmol siRNA or 4 μg plasmid using Lipofectamine 2000 (Thermo Fisher Scientific, Waltham, MA, USA) according to the manufacturer’s protocol when cells reached 60% confluence.

### 4.5. Serum Creatinine and Blood Urea Nitrogen Measurement

Serum creatinine and blood urea nitrogen levels were measured by an automatic chemistry analyzer (CS-400B, Dirui, Jilin, China).

### 4.6. Renal Histology and Immunohistochemistry

Paraffin-embedded mouse kidney sections (4 μm) were prepared using standard procedures, including fixation, dehydration, waxing, and embedding. For HE staining, sections were deparaffinized, rehydrated, stained with hematoxylin and eosin sequentially, dehydrated, cleared, and mounted for microscopic observation. Tubular injury scores were assigned according to the percentage of affected tubules per microscopic field: 0, no tubular injury; 1, <25% tubules affected; 2, 25–50% tubules affected; 3, 50–75% tubules affected; and 4, >75% tubules affected. For IHC staining, deparaffinized and rehydrated sections were subjected to antigen retrieval, blocked with 10% donkey serum, and incubated with primary antibodies overnight at 4 °C, followed by secondary antibody incubation at room temperature, DAB chromogenic detection, hematoxylin counterstaining, dehydration, clearing, mounting, and microscopic imaging. Primary antibodies used were as follows: IL-1β (1:100, ab283818, Abcam, Cambridge, MA, USA), IL-18 (1:100, A24169PM, ABclonal, Wuhan, China), and DDX3X (1:100, A5637, ABclonal, China).

### 4.7. Western Blotting

Total protein was extracted using RIPA lysis buffer containing protease and phosphatase inhibitors (Beyotime, Shanghai, China). Protein concentration was determined using a BCA Protein Assay Kit (Thermo Fisher Scientific, USA). Equal amounts of protein (20–60 μg) were separated by 8–12% SDS-PAGE and transferred onto PVDF membranes. Membranes were blocked with 5% non-fat milk for 60 min at room temperature and incubated overnight at 4 °C with primary antibodies. After washing with TBST, membranes were incubated with HRP-conjugated secondary antibodies for 1 h at room temperature. After TBST washing, bands were visualized using an ECL detection system (Abbkine, Wuhan, China).

Primary and secondary antibodies are listed below: G3BP1 (1:3000, 13057-2-AP, Proteintech, Wuhan, China), PARG (1:1000, 27808-1-AP, Proteintech, China), NGAL (1:1000, ab63929, Abcam, USA), NLRP3 (1:1000, ab214185, Abcam, USA), GSDMD-N (1:1000, 20770-1-AP, Proteintech, China), Cleaved-caspase-1 (1:1000, 22915-1-AP, Proteintech, China). For cleaved-caspase-1, only the 20 kDa fragment was quantified. DDX3X (1:1000, A5637, ABclonal, China), β-actin (1:5000, AC038, ABclonal, China), GAPDH (1:10,000, 10494-1-AP, Proteintech, China), HRP-conjugated goat anti-rabbit secondary antibody (1:8000, SA00001-2, Proteintech, China), and HRP-conjugated goat anti-mouse secondary antibody (1:8000, SA00001-1, Proteintech, China).

### 4.8. Co-IP

Cells were lysed in IP lysis buffer (Beyotime, China) supplemented with protease inhibitors (Beyotime, China) on ice for 30 min. Lysates were centrifuged, and the supernatant (1 mg total protein) was incubated with specific primary antibodies (1 μg) or control IgG at 4 °C for 6 h with gentle rotation. Pre-washed protein A/G magnetic beads (Beyotime, China) were added to the immune complexes and incubated overnight at 4 °C. Beads were washed five times with IP lysis buffer, boiled in SDS loading buffer (Servicebio, Wuhan, China), and subjected to Western blotting as described above. The primary and secondary antibodies are listed below: G3BP1 (1:1000, 13057-2-AP, Proteintech, China), NLRP3 (1:1000, ab214185, Abcam, USA), PARG (1:1000, 27808-1-AP, Proteintech, China), and DDX3X (1:1000, A5637, ABclonal, China). PARylation levels were detected using an anti-PAR antibody (1:100, sc-56198, Santa Cruz, Santa Cruz, CA, USA) and a goat anti-rabbit IgG (H+L) cross-adsorbed secondary antibody (1: 2000, M21006S, Abmart, Shanghai, China).

### 4.9. Immunofluorescence Staining

For tissue immunofluorescence staining, kidney paraffin sections (4 μm) were deparaffinized and subjected to antigen retrieval in EDTA buffer (pH 9.0) at 90 °C for 30 min. For cell staining, cells cultured on coverslips were fixed with cold methanol for 15 min and permeabilized with 0.5% Triton X-100 for 20 min at room temperature. After PBS washing, samples were blocked with 10% donkey serum for 1 h at room temperature and incubated with primary antibodies overnight at 4 °C. After washing, sections were incubated with fluorophore-conjugated secondary antibodies for 1 h at room temperature. Nuclei were counterstained with DAPI (1:1000, Beyotime, China). Images were captured using a fluorescence microscope (Nikon, Tokyo, Japan). Images were acquired using identical exposure settings within each experiment, and identical threshold parameters were applied for quantitative analysis. Quantification of SG-related parameters was performed using ImageJ (version 1.53c). SG-positive cells were defined as cells containing discrete G3BP1-positive cytoplasmic puncta above background fluorescence. Cells showing only diffuse cytoplasmic G3BP1 staining without distinct puncta were not considered SG-positive.

The primary and secondary antibodies are listed as follows: G3BP1 (1:200, 66486-1-Ig, Proteintech, China), AQP1 (1:100, 20333-1-AP, Proteintech, China), THP (1:100, A22544, ABclonal, China), AQP2 (1:100, A16209, ABclonal, China), TIA-1 (1:40, sc-166247, Santa Cruz, USA), NLRP3 (1:200, ab214185, Abcam, USA), DDX3X (1:200, A5637, ABclonal, China), Alexa Fluor 488 (1:300, A-11008, Thermo Fisher Scientific, USA), and Alexa Fluor 594 (1:300, A-11005, Thermo Fisher Scientific, USA).

### 4.10. Quantitative Real-Time PCR (qRT-PCR)

Total RNA was isolated from HK-2 cells by using a TRIzol RNA isolation system (Invitrogen, Carlsbad, CA, USA). The cDNA was synthesized from 2 µg of total RNA in a 20 µL reaction mixture containing AMV reverse transcriptase, random primers, and oligo primers at 42 °C for 60 min. qRT-PCR was performed using a Platinum SYBR Green qPCR Super Mix-UDG kit (Invitrogen, USA) in the QuantStudio 1 Real-Time PCR System (Thermo Fischer Scientific, USA). Analysis of gene expression was performed employing the 2−(ΔΔCt) method. Primer sequences were as follows: Parg (Human): Forward 5′-TGCCAGAGAGTCCATTGTCA-3′; Reverse 5′-CGGGTTCACTTTCCTTCTCA-3′. Gapdh (Human): Forward 5′-CGGAGTCAACGGATTTGGTCGTAT-3′; Reverse 5′-AGCCTTCTCCATGGTGGTGAAGAC-3′.

### 4.11. ELISA

Concentrations of IL-1β and IL-18 in cell culture supernatants were measured using a Human IL-1β Assay Kit (0181H1, HY cezmbio, Wuhan, China) and a Human IL-18 Assay Kit (0139H1, HY cezmbio, China), respectively, following the manufacturer’s protocols. The absorbance at 450 nm was detected using a microplate reader.

### 4.12. Cell Viability Assay

Cell viability was measured using a Cell Counting Kit-8 (CCK-8; C0038, Beyotime, China) assay, according to the manufacturer’s instructions. Briefly, HK-2 cells were seeded in 96-well plates and subjected to the indicated treatments. At the end of incubation, 10 μL of CCK-8 solution was added to each well, and cells were incubated at 37 °C for 1 h. The absorbance at 450 nm was recorded using a microplate reader.

### 4.13. LDH Release Assay

LDH release was determined using an LDH Cytotoxicity Assay Kit (C0016, Beyotime, China). Cell culture supernatants were collected and incubated with the reaction mixture according to the manufacturer’s instructions. The absorbance at 490 nm was measured using a microplate reader.

### 4.14. Caspase-1 Activity Assay

The caspase-1 activity was detected using a caspase-1 activity assay kit (C1101, Beyotime, China) according to the manufacturer’s instructions. Briefly, following the indicated treatments, HK-2 cells were collected and lysed using the provided lysis buffer on ice. Cell lysates were centrifuged at 16,000 *g* for 15 min at 4 °C, and the supernatants were collected for analysis. Equal amounts of protein samples were incubated with reaction buffer containing the caspase-1-specific substrate YVAD-AFC at 37 °C for 60–120 min. When the solution showed an obvious yellow p-nitroaniline (pNA) color, the reaction was stopped. The absorbance at 405 nm was measured using a microplate reader.

### 4.15. Statistical Analysis

Data are presented as the mean ± standard deviation (SD). Statistical analyses were performed with GraphPad Prism software (version 8.0). Differences between the two groups were analyzed using the Student’s *t*-test. Comparisons among multiple groups were performed using one-way ANOVA or two-way ANOVA followed by Tukey’s post hoc test. A *p*-value < 0.05 was considered statistically significant. All experiments were repeated at least three times.

## 5. Conclusions

This study demonstrates that SGs are predominantly formed in RTECs during AKI and contribute to renal protection. PARG-mediated PAR metabolism regulates SG dynamics, and PARG inhibition delays SG disassembly to promote SG persistence and enhance cellular adaptation. Mechanistically, persistent SGs regulate DDX3X availability and reduce DDX3X-associated NLRP3 inflammasome activation, thereby attenuating RTEC pyroptosis and renal injury. These findings reveal a novel mechanism of SG-mediated renoprotection and highlight SG dynamics as a potential therapeutic target in AKI.

## Data Availability

The raw data supporting the conclusions of this article will be made available by the authors upon request.

## Supplementary Materials

The following supporting information can be downloaded at: https://www.mdpi.com/article/10.3390/ijms27167192/s1.

## Abbreviations

AKI	Acute kidney injury
RTECs	Renal tubular epithelial cells
SGs	Stress granules
PARylation	Poly(ADP-ribosyl)ation
PARG	Poly(ADP-ribose) glycohydrolase
NLRP3	NOD-like receptor protein 3
GSDMD	Gasdermin-D
PTMs	Post-translational modifications
PARPs	Poly(ADP-ribose) polymerases
DDX3X	DEAD-box helicase 3 X-linked
AAV	Adeno-associated virus
HE	Hematoxylin and eosin
IHC	Immunohistochemical
Co-IP	Co-Immunoprecipitation
qRT-PCR	Quantitative real-time PCR
ELISA	Enzyme-linked immunosorbent assay
LDH	Lactate dehydrogenase
