# Supplementary material for: Delaying Stress Granule Disassembly by PARG Inhibition Attenuates Renal Tubular Cell Pyroptosis in Acute Kidney Injury
Source: Int J Mol Sci. 2026 Aug 12;27(16):7192. doi: 10.3390/ijms27167192 (PMC13513823; doi:10.3390/ijms27167192)
Supplement: Supplementary file 1 [file ijms-27-07192-s001.zip › ijms-4391860-supplementary.pdf]

**Sample ID**	**Group**	**Age**	**Sex**	**Diagnosis**	**Biopsy indication**	**AKI stage**
AKI#1	AKI	38	Male	Drug-associated AKI	Persistent AKI with incomplete renal recovery	AKI Stage 3
AKI#2	AKI	40	Male	Drug-associated AKI	Persistent AKI with incomplete renal recovery	AKI Stage 3
AKI#3	AKI	35	Female	Acute tubular injury	Unexplained AKI	AKI Stage 3
AKI#4	AKI	47	Male	Acute tubular injury	Unexplained AKI	AKI Stage 3
Donor#1	Control	42	Male	Donor kidney	Transplant evaluation	NA
Donor#2	Control	39	Male	Donor kidney	Transplant evaluation	NA
Donor#3	Control	52	Male	Donor kidney	Transplant evaluation	NA

Supplementary Table S1 Clinical characteristics of human kidney samples

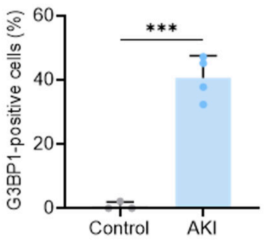

Figure S1 Quantification of G3BP1-positive tubular epithelial cells in human kidney samples

Quantification of the percentage of G3BP1-positive tubular epithelial cells in individual human renal biopsy samples. n = 3 for the control kidney from donors and 4 for the AKI patients. ***$P < 0.001$ vs. Control. Data were analyzed by Student's *t*-tests.

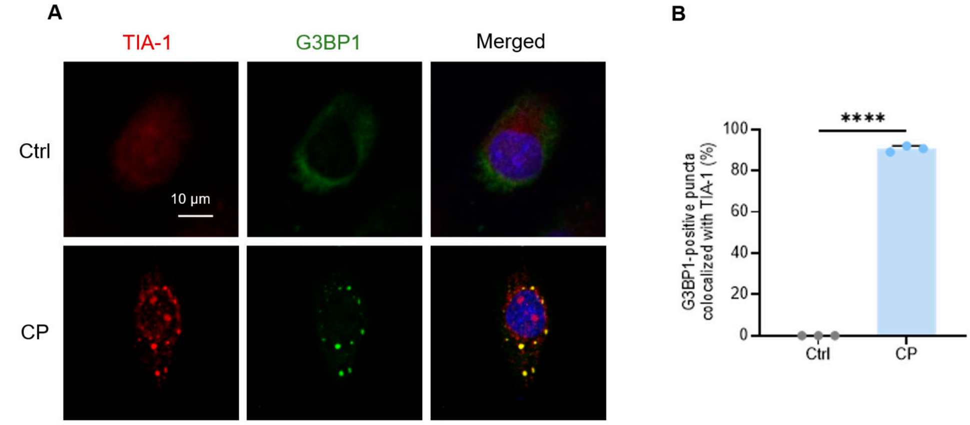

Figure S2 Validation of G3BP1-positive puncta as SGs by TIA-1 colocalization in HK-2 cells (A) Immunofluorescence co-localization of TIA-1 (red) and G3BP1 (green) in HK-2 cells. Scale bar: 10 μm. (B) Quantification of the percentage of G3BP1-positive puncta that were also positive for TIA-1. n=3 biological replicates per group. ****P < 0.0001 vs. Ctrl. Data were analyzed by Student's t-tests (B).

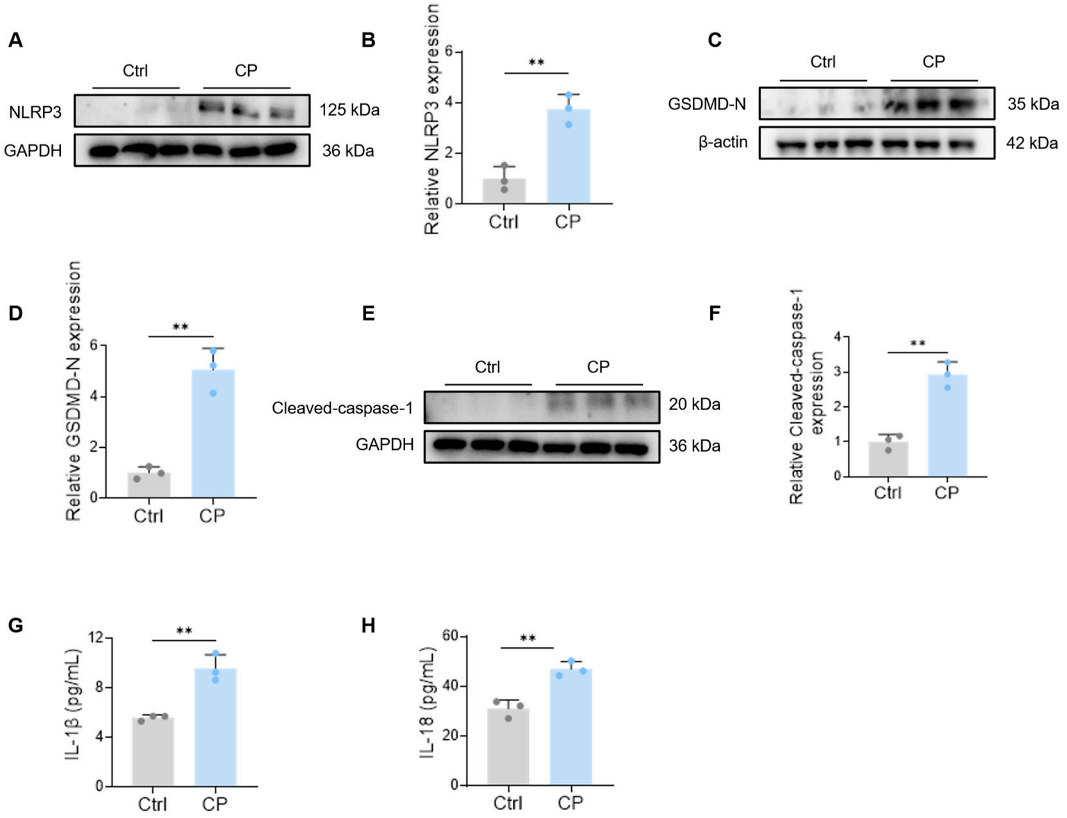

Figure S3 Cisplatin treatment promotes pyroptosis in HK-2 cells
(A-B) Representative western blot images and quantification of NLRP3 expression in HK-2 cells. n=3 biological replicates per group. $^{**}P < 0.01$ vs. Ctrl. (C-D) Representative western blot images and quantification of GSDMD-N expression in HK-2 cells. n=3 biological replicates per group. $^{**}P < 0.01$ vs. Ctrl. (E-F) Representative western blot images and quantification of Cleaved-caspase-1 expression in HK-2 cells. n=3 biological replicates per group. $^{**}P < 0.01$ vs. Ctrl. (G) ELISA of IL-1β in the culture supernatant of HK-2 cells. n=3 biological replicates per group. $^{**}P < 0.01$ vs. Ctrl. (H) ELISA of IL-18 in the culture supernatant of HK-2 cells. n=3 biological replicates per group. $^{**}P < 0.01$ vs. Ctrl. Data were analyzed by Student's *t*-tests (B, D, F, G, H).

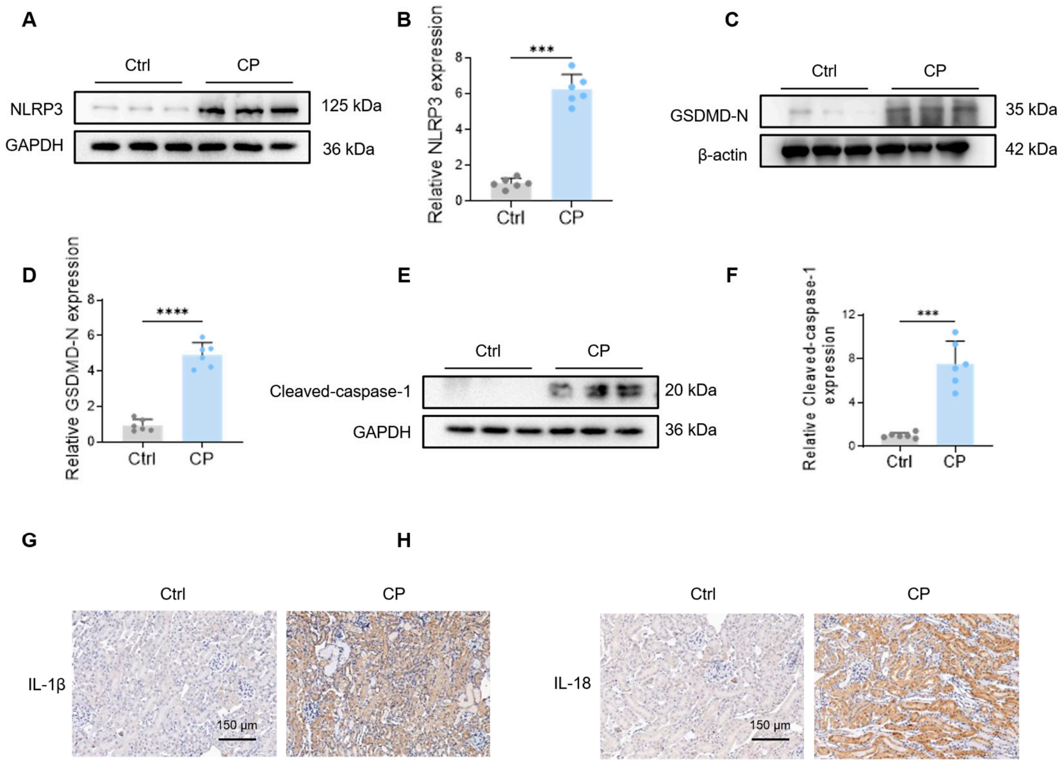

Figure S4 Cisplatin administration promotes renal pyroptosis in AKI mice
(A-B) Representative western blot images and quantification of NLRP3 expression in renal tissues. n=6 biological replicates per group. ****P* < 0.001 vs. Ctrl. (C-D) Representative western blot images and quantification of GSDMD-N expression in renal tissues. . n=6 biological replicates per group. *****P* < 0.0001 vs. Ctrl. (E-F) Representative western blot images and quantification of Cleaved-caspase-1 expression in renal tissues. . n=6 biological replicates per group. *****P* < 0.0001 vs. Ctrl. (G) IHC staining of IL-1β in renal sections of renal sections. Scale bar: 150 μm. (H) IHC staining of IL-18 in renal sections of renal sections. n=6 biological replicates per group. Scale bar: 150 μm. Data were analyzed by Student's *t*-tests (B, D, F).

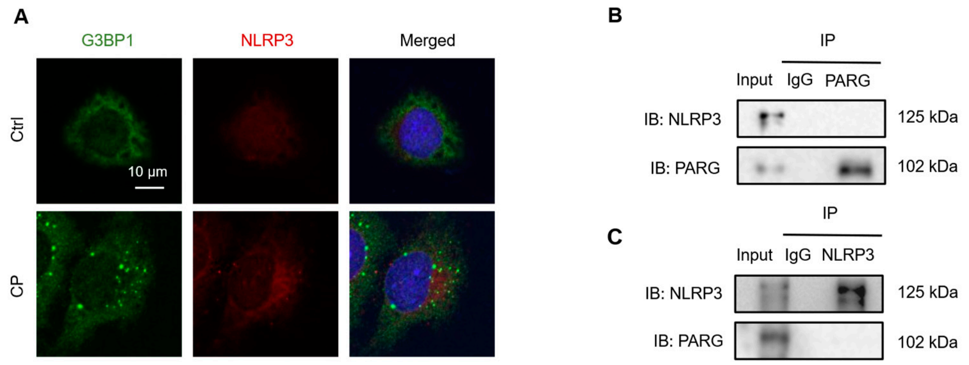

Figure S5 NLRP3 is not sequestered by SGs and does not interact with PARG
(A) Immunofluorescence co-localization of G3BP1 (green) and NLRP3 (red) in HK-2 cells. Scale bar: 10 μm. (B-C) Co-IP assays demonstrating the interaction between PARG and NLRP3 in cisplatin-stimulated HK-2 cells.

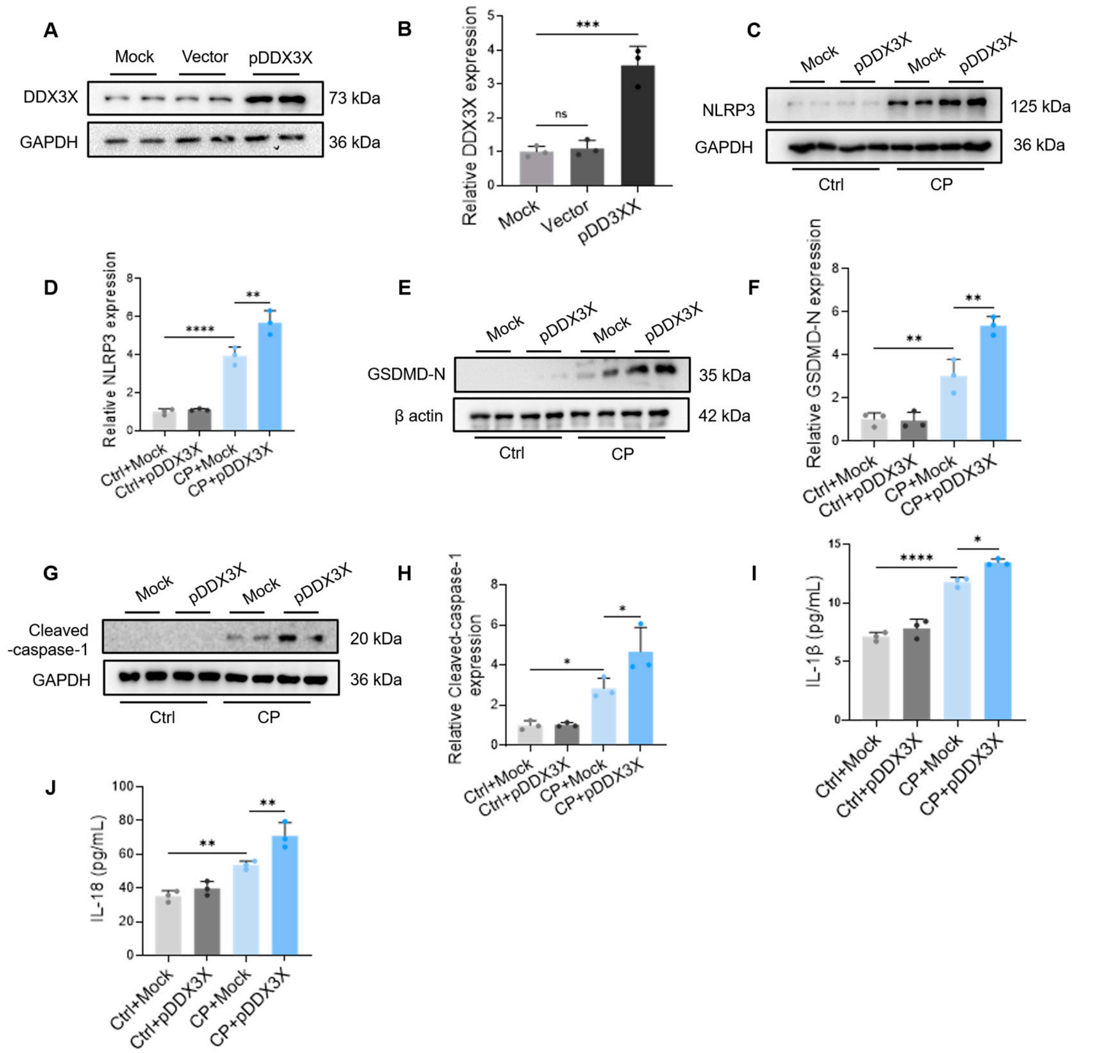

Figure S6 Overexpression of DDX3X promotes cisplatin-induced pyroptosis in HK-2 cells (A-B) Representative western blot images and quantification of DDX3X expression in HK-2 cells transfected with empty vector (Mock) or DDX3X-overexpressing plasmid (pDDX3X). n=3 biological replicates per group. ns, not significant vs. Vector. ***$P < 0.001$ vs. pDDX3X. (C-D) Representative western blot images and quantification of NLRP3 expression in HK-2 cells. n=3 biological replicates per group. **$P < 0.01$ vs. CP+ pDDX3X, ****$P < 0.0001$ vs. Ctrl+Mock. (E-F) Representative western blot images and quantification of GSDMD-N expression in HK-2 cells. n=3 biological replicates per group. **$P < 0.01$ vs. CP+ pDDX3X/Ctrl+Mock. (G-H) Representative western blot images and quantification of Cleaved-caspase-1 expression in HK-2 cells. n=3 biological replicates per group. *$P < 0.05$ vs. CP+ pDDX3X/Ctrl+Mock. (I) ELISA of IL-1β in the culture supernatant of HK-2 cells. n=3 biological replicates per group. **$P < 0.01$ vs. CP+ pDDX3X, ****$P < 0.0001$ vs. Ctrl+Mock. (J) ELISA of IL-18 in the culture supernatant of HK-2 cells. n=3 biological replicates per group. **$P < 0.01$ vs. CP+ pDDX3X/Ctrl+Mock. Data were analyzed by one-way ANOVA with Tukey's multiple comparisons (B, D, F, H, I, J).

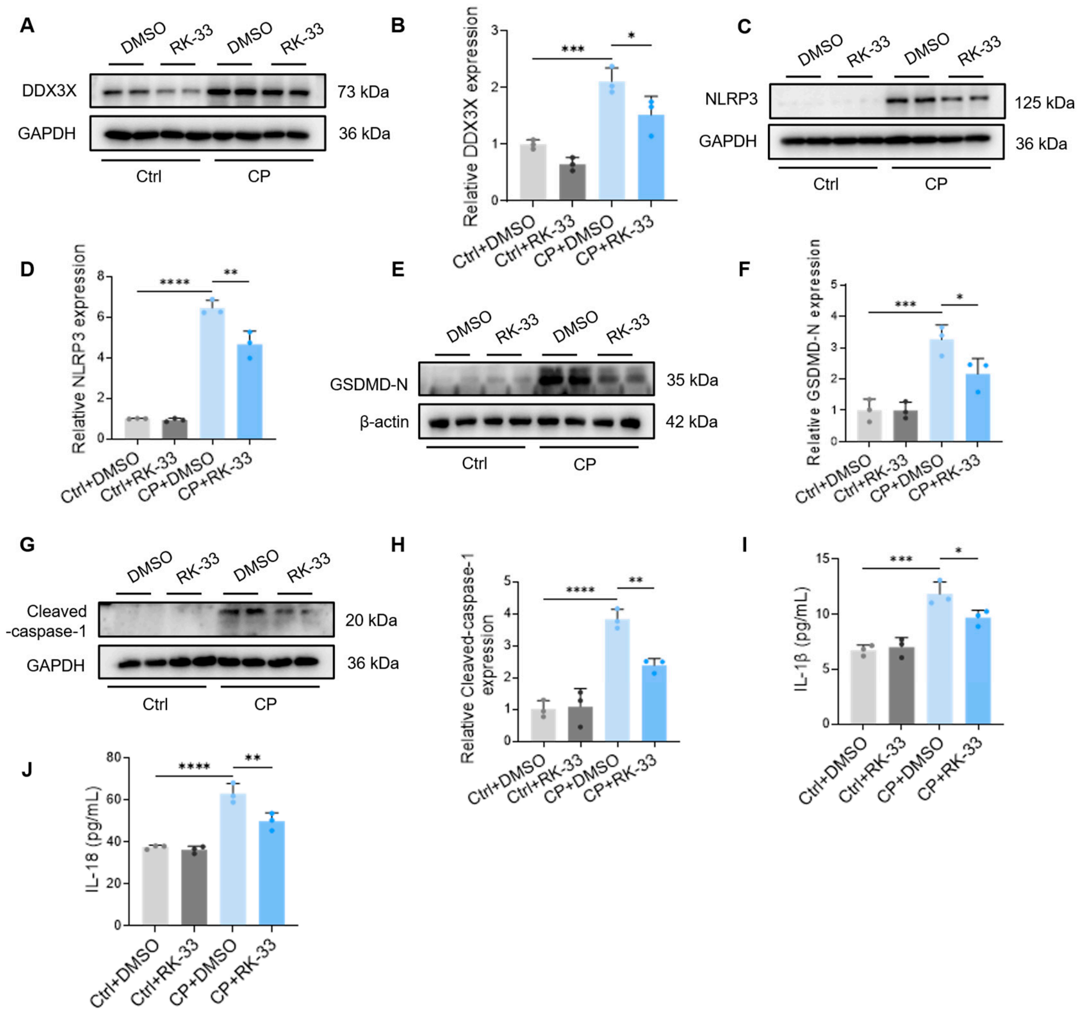

Figure S7 DDX3X inhibition by RK-33 suppresses cisplatin-induced pyroptosis in HK-2 cells

(A-B) Representative western blot images and quantification of DDX3X expression in HK-2 cells treated with DMSO (vehicle control) or RK-33. n=3 biological replicates per group. **P* < 0.05 vs. CP+ RK33, ****P* < 0.001 vs. Ctrl+DMSO. (C-D) Representative western blot images and quantification of NLRP3 expression in HK-2 cells. n=3 biological replicates per group, ***P* < 0.01 vs. CP+ RK33, *****P* < 0.0001 vs. Ctrl+DMSO. (E-F) Representative western blot images and quantification of GSDMD-N expression in HK-2 cells. n=3 biological replicates per group. **P* < 0.05 vs. CP+ RK33, ****P* < 0.001 vs. Ctrl+DMSO.

(G-H) Representative western blot images and quantification of Cleaved-caspase-1 expression in HK-2 cells. n=3 biological replicates per group, ***P* < 0.01 vs. CP+ RK33, *****P* < 0.0001 vs. Ctrl+DMSO. (I) ELISA of IL-1β in the culture supernatant of HK-2 cells. n=3 biological replicates per group, **P* < 0.05 vs. CP+ RK33, ****P* < 0.001 vs. Ctrl+DMSO. (J) ELISA of IL-18 in the culture supernatant of HK-2 cells. n=3 biological replicates per group, ***P* < 0.01 vs. CP+ RK33, *****P* < 0.0001 vs. Ctrl+DMSO. Data were analyzed by one-way ANOVA with Tukey's multiple comparisons (B, D, F, H, I, J).

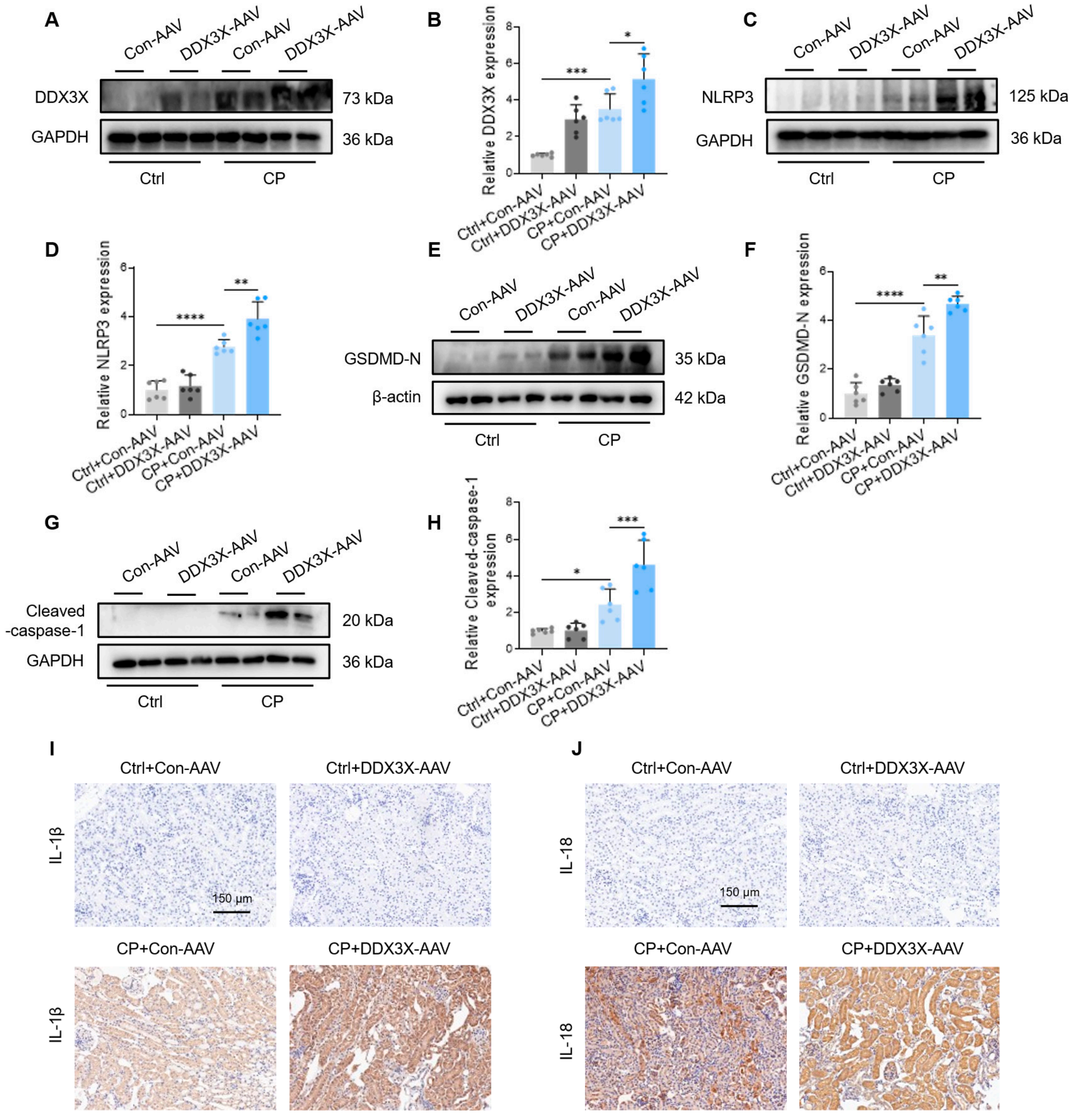

Figure S8 Renal DDX3X overexpression exacerbates cisplatin-induced pyroptosis in AKI mice

(A-B) Representative western blot images and quantification of DDX3X expression in renal tissues from mice injected with control AAV (Con-AAV) or DDX3X-overexpressing AAV (DDX3X-AAV). n=6 biological replicates per group, **P* < 0.05 vs. CP+DDX3X-AAV, ****P* < 0.001 vs. Ctrl+Con-AAV. (C-D) Representative western blot images and quantification of NLRP3 expression in renal tissues. n=6 biological replicates per group, ***P* < 0.01 vs. CP+DDX3X-AAV, *****P* < 0.0001 vs. Ctrl+Con-AAV. (E-F) Representative western blot images and quantification of GSDMD-N expression in renal tissues. n=6 biological replicates per group, ***P* < 0.01 vs. CP+DDX3X-AAV, *****P* < 0.0001 vs. Ctrl+Con-AAV. (G-H) Representative western blot images and quantification of Cleaved-caspase-1 expression in renal tissues. n=6 biological replicates per group, **P* < 0.05 vs. CP+DDX3X-AAV, ****P* < 0.001 vs. Ctrl+Con-AAV. (I) IHC staining of IL-1β in renal sections of renal sections. Scale bar: 150 μm. (J) IHC staining of IL-18 in renal sections of renal sections. Scale bar: 150 μm. Data were analyzed by one-way ANOVA with Tukey's multiple comparisons (B, D, F, H).

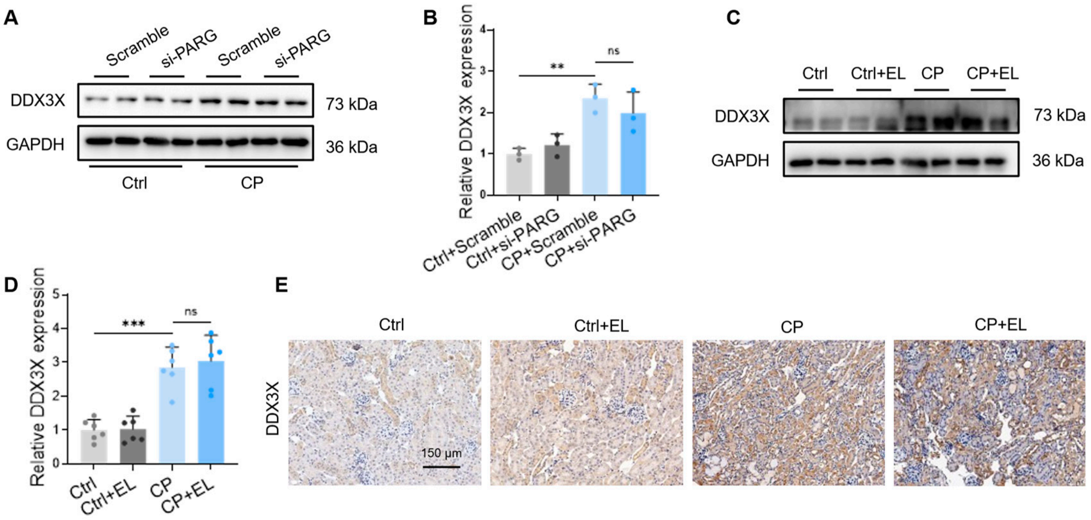

Figure S9 DDX3X expression is unaffected by SGs

(A-B) Representative western blot images and quantification of DDX3X expression in HK-2 cells. n=3 biological replicates per group. ns, not significant vs. CP+si-PARG, ***P* < 0.01 vs. Ctrl+Scramble. (C-D) Representative western blot images and quantification of DDX3X expression in renal. . n=3 biological replicates per group. ns, not significant vs. CP+EL, ****P* < 0.001 vs. Ctrl. (E) IHC staining of DDX3X of renal sections. Scale bar: 150 μm. Data were analyzed by one-way ANOVA with Tukey's multiple comparisons (B, D).
